# Recent Advances in Flexible and Wearable Gas Sensors Harnessing the Potential of 2D Materials

**DOI:** 10.1002/smsc.202500025

**Published:** 2025-06-30

**Authors:** Azmira Jannat, Md Mehdi Masud Talukder, Zhong Li, Jian Zhen Ou

**Affiliations:** ^1^ Key Laboratory of Advanced Technologies of Materials, Ministry of Education, School of Materials Science and Engineering Southwest Jiaotong University Chengdu 610031 China; ^2^ School of Engineering RMIT University Melbourne Victoria 3000 Australia

**Keywords:** 2D materials, environmental monitoring, flexible gas sensors, healthcare applications, hybrid materials, MXenes, wearable gas sensors

## Abstract

In the Internet of Things era, flexible and wearable gas sensors are increasingly vital for real‐time monitoring in healthcare, environmental safety, and industrial security. These sensors detect hazardous gases at room temperature and seamlessly integrate with clothing and portable devices. The 2D materials, including transition metal dichalcogenides, black phosphorus, MXenes, graphene and its derivatives, and metal–organic frameworks, stand out due to their exceptional electrical, mechanical, and physicochemical properties, such as large surface areas, high carrier mobility, and intrinsic flexibility. This review summarizes recent advancements in designing, fabricating, and applying 2D‐material‐based flexible gas sensors. It highlights how engineering approaches like defect creation, composite formation, and surface functionalization significantly enhance sensor sensitivity, selectivity, and response times. Comparative performance data across various material families are presented, alongside effective strategies for integrating 2D materials onto diverse flexible substrates such as polymers, textiles, and paper, emphasizing durability under mechanical stress. The review critically addresses current challenges, including large‐scale manufacturing, long‐term stability, and interference from ambient humidity. Furthermore, it explores innovative solutions like self‐healing sensors, artificial intelligence‐driven sensor arrays, in situ surface passivation, and multisensor platforms coupled with machine learning algorithms, offering valuable insights for advancing next‐generation wearable gas‐sensing technologies.

## Graphical abstract

This review explores cutting‐edge advancements in 2D‐material‐based flexible gas sensors for real‐time detection in health, environmental, and industrial settings. It discusses material innovations, fabrication techniques, and performance enhancements through defect engineering and functionalization. Integration with flexible substrates, durability challenges, and emerging solutions such as AI‐driven platforms and self‐healing materials are highlighted, guiding next‐generation wearable gas sensor development.

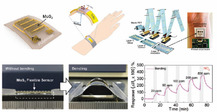

## 1 Introduction

Environmental monitoring has become increasingly critical due to escalating air pollution and its detrimental effects on human health and ecosystems. Toxic gases and volatile organic compounds (VOCs) released from industrial processes, urban activities, and natural events significantly contribute to environmental degradation and health hazards. Gas sensing technologies play a pivotal role by enabling real‐time detection and quantification of hazardous gases, facilitating timely interventions to mitigate their adverse effects. According to the World Health Organization (WHO), air pollution (ambient and household combined) is associated with around 6.7 million premature deaths annually.^[^ ^1^ ^]^ Thus, the deployment of effective gas sensors is essential for air quality monitoring, early hazard detection, and compliance with regulatory standards.^[^ ^2^ ^]^ Furthermore, in industrial settings, wearable gas detectors can alert personnel to gas leaks or unsafe conditions, minimizing occupational risks.^[^ ^3^, ^4^ ^]^ Certain gases, such as nitrogen dioxide (NO_2_), carbon monoxide (CO), ammonia (NH_3_), sulfur dioxide (SO_2_), and methane (CH_4_), pose significant threats to human health and the environment. NO_2_ is associated with respiratory and cardiovascular diseases, while CO, being colorless and odorless, is highly toxic even at low concentrations.^[^ ^5^, ^6^ ^]^ NH_3_, SO_2_, and CH_4_ contribute to environmental degradation through acid rain, greenhouse gas effects, and potential fire hazards in industrial contexts.^[^ ^7^, ^8^ ^]^ Detecting these gases at trace levels is crucial for ensuring environmental sustainability, public safety, and industrial hygiene.^[^ ^9^, ^10^ ^]^

Wearable gas sensors are of paramount importance as they bridge the gap between traditional sensing technologies and the need for real‐time, on‐the‐go applications. These devices address the inherent limitations of rigid, bulky, and stationary gas sensors, offering affordable, noninvasive, and user‐friendly alternatives for applications spanning environmental monitoring, public safety, health diagnostics, and food quality control. In environmental monitoring, wearable gas sensors empower individuals to assess air quality in real time, enabling early detection of hazardous gases such as carbon monoxide and nitrogen dioxide. This contributes significantly to public health by mitigating exposure to air pollutants, a pressing issue in urban and industrialized areas.^[^ ^11^, ^12^ ^]^ In public safety, wearable gas sensors are indispensable for industries like mining, chemical processing, and firefighting, where toxic gas leaks pose serious threats. Their portability and rapid response times allow for immediate detection, enhancing workplace safety and preventing catastrophic incidents.^[^ ^13^, ^14^ ^]^ In healthcare, wearable gas sensors have revolutionized diagnostics by enabling the noninvasive monitoring of biomarkers in exhaled breath. VOCs in breath serve as indicators for conditions like diabetes, asthma, and lung infections. Continuous monitoring facilitates early detection and timely medical intervention, making these sensors crucial for personalized healthcare.^[^ ^2^, ^12^ ^]^ Wearable gas sensors also play a pivotal role in food quality control, detecting spoilage markers and gas emissions from contaminated food products. Their integration into packaging solutions ensures compliance with safety standards and reduces food wastage.^[^ ^15^ ^]^

Over the past two decades, wearable devices have undergone substantial evolution, with innovations like the Bluetooth headset in 2002, Nike+ in 2006, FitBit in 2008, and the Apple Watch in 2015 marking significant milestones in their development.^[^ ^16^, ^17^, ^18^, ^19^ ^]^ These advancements have transformed wearable technologies into critical tools for real‐time monitoring in healthcare and safety applications. Gas sensors designed for wearables are uniquely capable of continuous monitoring, enabling real‐time tracking of vital health parameters. This capability facilitates early detection of potential health risks, enhancing timely medical interventions. Beyond healthcare, wearable gas sensors have found applications in environmental surveillance,^[^ ^20^, ^21^ ^]^ activity monitoring,^[^ ^22^ ^]^ workplace safety,^[^ ^23^ ^]^ and human–machine interactions.^[^ ^23^, ^24^ ^]^ Their versatility and adaptability underscore their role as pivotal tools in addressing modern challenges across diverse domains.^[^ ^25^, ^26^ ^]^

Traditional gas sensor technologies, including calorimetric, thermoelectric, optical, and chromatographic methods, rely on bulky, complex equipment, and skilled operators, making them unsuitable for wearable applications.^[^ ^4^ ^]^ While electronic transducing sensors offer advantages like miniaturization and adaptability, their rigid substrates (e.g., silicon, glass, ceramics) and high operating temperatures, often exceeding 200 °C, restrict their use to controlled environments and stationary setups.^[^ ^5^ ^]^ Additionally, conventional sensors often lack the mechanical adaptability to withstand bending, twisting, and stretching, making them impractical for integration into dynamic and conformal systems like wearable electronics.^[^ ^4^ ^]^ These limitations highlight the need for innovative approaches, such as 2D materials, to overcome the challenges of implementing gas sensors in wearable formats.

Wearable gas sensors emerge as a promising solution by addressing the shortcomings of traditional technologies, particularly their lack of flexibility and adaptability. Designed to maintain functionality under mechanical deformation such as bending, stretching, or twisting these sensors overcome the rigid constraints of conventional counterparts. They are lightweight, cost‐effective, and compact, making them ideal for wearable applications.^[^ ^3^, ^27^ ^]^ Furthermore, wearable gas sensors can be categorized into three types based on their integration: “on‐body,” “in‐clothing,” and “accessory” sensors. “On‐body” sensors adhere to the skin and require biocompatible, non‐irritating substrates. “In‐clothing” sensors are embedded within textiles and must withstand regular washing cycles, while “accessory” sensors, such as wristbands or watches, are designed for portability and ease of use.^[^ ^28^, ^29^ ^]^ These categories reflect the versatility of wearable gas sensors, which seamlessly integrate into everyday life while maintaining functionality and comfort.

The realization of wearable gas sensors relies on selecting appropriate components, including substrates, electrodes, and sensing materials. Polymer‐based substrates, such as polyimide (PI), polyethylene terephthalate (PET), and polydimethylsiloxane (PDMS), are particularly desirable due to their flexibility, mechanical durability, and chemical stability.^[^ ^3^ ^]^ These substrates enable sensors to adapt to various applications, ensuring compatibility with wearable designs. Conductive nanomaterials, including graphene, silver nanowires, and carbon nanotubes, serve as electrodes, providing excellent electrical performance without compromising flexibility. The integration of advanced fabrication techniques, such as inkjet printing and electrospinning, ensures scalable production while maintaining structural integrity and performance under mechanical stress.^[^ ^5^, ^30^ ^]^

Sensing materials are critical to the performance of flexible and wearable gas sensors. Traditional semiconductors, like silicon (Si), have been used for flexible devices by reducing thickness below a threshold.^[^ ^31^ ^]^ However, Si's brittleness limits reliability, while organic semiconductors suffer from low charge carrier mobility and high operational voltages, impacting sensitivity and complicating design.^[^ ^32^, ^33^ ^]^ Polycrystalline Si offers better mobility but is hindered by high leakage currents and production costs, making it less suitable for flexible electronics.^[^ ^34^ ^]^ These challenges underscore the need for alternative materials in wearable device technology.

In recent years, 2D materials have attracted tremendous interest for advanced electronics due to their exceptional electrical, mechanical, and chemical properties.^[^ ^35^ ^]^ These materials, including graphene, transition metal dichalcogenides (TMDs), black phosphorus (BP), hexagonal boron nitride (hBN), nitride nanocomposite materials, and MXenes, feature low power consumption, high charge mobility, stretchability, and mechanical stability, making them ideal for flexible devices.^[^ ^2^, ^36^, ^37^, ^38^ ^]^ Their large surface‐to‐volume ratios provide abundant adsorption sites including vacancy, edge, defects, and basal planes enabling unparalleled chemical sensing capabilities. For example, graphene, the prototypical 2D material, can undergo notable resistivity changes upon gas adsorption even at room temperature (RT), and it can be integrated onto textiles or flexible electronics without performance loss.^[^ ^7^ ^]^ Beyond single‐component materials, composites such as graphene oxide (GO)/polymer blends or metal–organic framework (MOF)‐on‐2D hybrids have been developed to synergistically improve selectivity and stability.^[^ ^15^, ^39^ ^]^

Recent advancements have expanded 2D materials’ applications to include foldable displays, chemical sensors, and photodetectors.^[^ ^40^, ^41^, ^42^ ^]^ Their ability to detect gases at RT eliminates the need for microheaters, reducing power consumption from hundreds of milliwatts to microwatts, establishing them as energy‐efficient alternatives for modern sensors.^[^ ^43^, ^44^ ^]^ Additionally, 2D materials possess exceptional attributes, including lightweight, cost‐effectiveness, transparency, and flexibility or stretchability, which align perfectly with the essential requirements for flexible and wearable sensors. Their outstanding mechanical flexibility is a key advantage, enabling them to maintain high sensing performance even under mechanical stresses like bending, stretching, or molding, without experiencing degradation or deformation. Furthermore, advanced surface and interface engineering techniques, such as the decoration of 2D materials with metal nanoparticles (NPs) or organic molecules and their integration with other materials, play a crucial role in enhancing gas sensing capabilities on flexible and wearable sensing platforms.^[^ ^3^ ^]^ These developments highlight the transformative potential of 2D materials in addressing limitations of traditional technologies and driving innovations in flexible, high‐performance sensing solutions.

This review comprehensively explores critical and comparative perspective specifically on flexible and wearable gas sensors enabled by 2D materials. This review not only summarizes recent examples but also compares the performance of different 2D material systems under similar conditions, identifies gaps in knowledge such as long‐term stability and multigas sensing challenges, and proposes strategies to address these issues. In particular, a structured framework is introduced in Section 3.7 to guide readers on material selection and sensor design tailored to various application domains, including environmental air quality monitoring, healthcare (e.g., breath analysis), and industrial safety, based on the unique requirements of each field. Additionally, insights from theoretical studies and in situ experiments are incorporated to elucidate the underlying gas sensing mechanisms, thereby bridging the gap between empirical findings and fundamental understanding. Following this introduction, Section 2 of the review covers the fundamental components of wearable gas sensors and common fabrication techniques, establishing a foundation for understanding how 2D materials can be integrated into flexible devices. Section 3 constitutes the core of the review, presenting a discussion on various classes of 2D materials including graphene and its derivatives, TMDs, BP, MXenes, MOFs, and their nanoengineered composites for gas sensing applications. Detailed examples of sensor performance are provided, with each subsection offering quantitative data (e.g., sensitivity, response time, and detection limits) from representative studies to facilitate cross‐comparison. Section 4 addresses the current limitations and challenges associated with the real‐world application of these sensors, such as scaling up production, ensuring stability and repeatability, and managing humidity interference, while also surveying emerging solutions, including novel materials and innovative device architectures. Finally, Section 5 presents the conclusions and future prospects, suggesting key areas for further research, including the development of self‐powered sensor systems, the enhancement of AI algorithms for gas sensor arrays, and the exploration of new 2D materials such as Janus structures and perovskite nanosheets. Through this comprehensive and up‐to‐date review, the aim is to inform and advance the development of next‐generation flexible and wearable gas sensors that fully harness the potential of 2D materials.

## 2 Fundamental Components and Fabrication Techniques of Wearable Chemical Sensing

A standard wearable gas sensor consists of a stretchable substrate and two key functional parts: a sensing material and a transduction unit.^[^ ^5^, ^45^ ^]^ The sensing material interacts with the analyte gases, leading to changes in its chemical and physical properties (e.g., electrical conductivity, permittivity, or work function), which are then converted into a readable signal, such as current (I), capacitance (C), resistance (R)/impedance (Z), voltage (V), or electrical potential (E), through the transduction unit.^[^ ^5^ ^]^ By employing different types of transducers, such as inter digitate electrode (IDE),^[^ ^46^ ^]^ resistors,^[^ ^47^ ^]^ capacitors,^[^ ^48^ ^]^ diodes,^[^ ^49^ ^]^ field‐effect transistors (FETs),^[^ ^50^ ^]^ the presence of gas analytes can be detected and quantified by observing the changes in measurable electrical signals. These signals are subsequently processed, including amplification, filtering, and analysis, to provide meaningful information regarding the type and concentration of gas analytes.^[^ ^5^ ^]^ Moreover, the choice of substrate plays a key role in the design of wearable devices. The following discussion will explore the various components that contribute to wearable gas sensor design in detail.

### 2.1 Flexible Substrates

Wearable gas sensors have been developed in response to the growing demand for environmental safety, health monitoring, public safety, security, and food quality assurance. Traditional gas sensor fabrication methods typically rely on rigid substrates such as glass,^[^ ^51^ ^]^ and indium tin oxide (ITO)‐coated glass,^[^ ^25^ ^]^ silicon,^[^ ^52^, ^53^ ^]^ and SiO_2_‐coated Si.^[^ ^54^, ^55^ ^]^ However, these substrates are unsuitable for flexible or stretchable applications. Wearable gas sensors, designed to be either applied directly to the skin or integrated into clothing and accessories, must be lightweight, compact, and user‐friendly. Along with being lightweight and cost‐effective, the substrates used in wearable electronics need to be flexible or stretchable. Materials such as commercially available polymers,^[^ ^56^ ^]^ tattoo papers,^[^ ^57^ ^]^ textile‐based platforms,^[^ ^58^ ^]^ masks,^[^ ^59^ ^]^ and gloves^[^ ^60^ ^]^ have all been explored as base substrates for wearable gas sensors.

#### 2.1.1 Polymer‐Based Substrates

Polymeric substrates like flexible PI,^[^ ^56^ ^]^ PDMS,^[^ ^61^ ^]^ PET,^[^ ^24^ ^]^ parylene,^[^ ^5^ ^]^ silk,^[^ ^62^ ^]^ acrylic,^[^ ^63^ ^]^ and Ecoflex^[^ ^47^ ^]^ are commonly used in wearable gas sensors due to their unique properties, which cater to different application needs.

**Figure 1:**
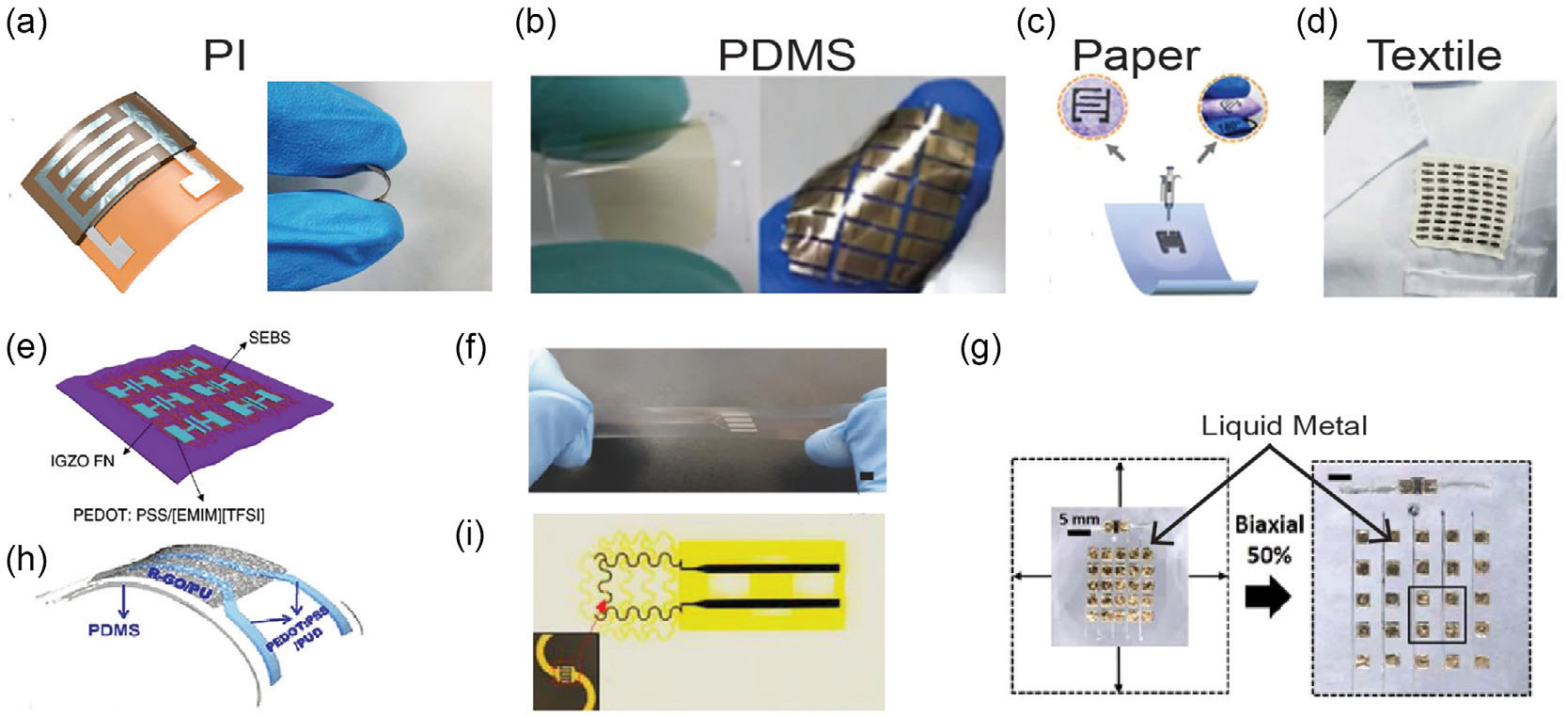
a) Schematic representation of a CuBr gas sensor, demonstrating its structural design. Flexibility is enhanced with a 100 nm‐thick CeO_2_ overlayer. Reproduced with permission.^[^ ^21^ ^]^ Copyright 2018, American Chemical Society. b) An all‐graphene electronic skin (e‐skin) sensor on a PDMS substrate, showcasing the device's adaptability and stretchability. Reproduced with permission.^[^ ^65^ ^]^ Copyright 2016, Wiley‐VCH. c) A flexible, paper‐based gas sensor integrated with a Ti_3_C_2_T_x_/WS_2_ layer, highlighting its innovative design and functionality for wearable applications. Reproduced with permission.^[^ ^73^ ^]^ Copyright 2023, American Chemical Society. d) Photographs of an array of gas sensors stitched onto a laboratory coat, illustrating the feasibility of wearable e‐textile‐based gas sensing. Reproduced with permission.^[^ ^62^ ^]^ Copyright 2018, American Chemical Society. e) A stretchable electrode made of poly(3,4‐ethylenedioxythiophene):poly(styrenesulfonate) (PEDOT:PSS) combined with the ionic liquid [EMIM][TFSI], deposited on a poly[styrene‐b‐(ethylene‐co‐butylene)‐b‐styrene] (SEBS) substrate, designed for indium–gallium–zinc oxide (IGZO) NF network resistors. Reproduced with permission. Copyright 2020, Nature Publishing Group. f) A composite electrode made of conducting silver nanowires (Ag NWs) embedded in an ionic thermoplastic polyurethane (i‐TPU) polymer electrolyte, optimized for VOC detection. Reproduced with permission.^[^ ^18^ ^]^ Copyright 2018, Wiley‐VCH. g) Liquid metal interconnections composed of Galinstan (a eutectic alloy of gallium (68.5%), indium (21.5%), and tin (10%) applied to Silbione/PDMS substrates, showcasing advanced flexibility and durability. Reproduced with permission.^[^ ^61^ ^]^ Copyright 2017, Nature Publishing Group. h) A stretchable electrode made of a PEDOT:PSS/polyurethane dispersion (PUD) nanocomposite on a PDMS substrate, specifically designed for humidity sensing. Reproduced with permission.^[^ ^23^ ^]^ Copyright 2017, Springer Nature. i) A serpentine gold (Au) interconnect sandwiched between two PI layers, designed to ensure robust mechanical and electrical performance. Reproduced with permission.^[^ ^227^ ^]^ Copyright 2019, Wiley‐VCH.

*PDMS‐based substrates* are extensively used in stretchable electronics because of their high elasticity (≈100%–1100%), flexibility, and biocompatibility, making them ideal for wearable sensors that conform to the skin. PDMS is particularly well‐suited for skin‐friendly devices due to its excellent stretchability, allowing it to maintain functionality even when subjected to large deformations. However, one major challenge with PDMS is its low surface energy, which leads to poor adhesion with other sensor components. To overcome this, treatments such as oxygen plasma, ultraviolet (UV) exposure, and chemical functionalization are applied to enhance surface adhesion.^[^ ^64^ ^]^ For instance, oxygen plasma treatment has been successfully used to fabricate highly stretchable e‐skin sensors (**Figure** **1**b), which demonstrate remarkable stability even after 10,000 deformation cycles at 40% strain.^[^ ^23^, ^65^ ^]^ These characteristics make PDMS a leading choice for stretchable sensor platforms, especially in “on‐body” applications.

*PI substrates* are widely utilized in wearable gas sensors due to their affordability, flexibility, and moderate stretchability.^[^ ^5^, ^49^ ^]^ PI is particularly valued for its exceptional bendability and high thermal stability (360–410 °C), making it well‐suited for integration with conventional microelectronics fabrication techniques (Figure 1a).^[^ ^21^ ^]^ PI‐based gas sensors exhibit impressive mechanical durability, as demonstrated by their ability to maintain performance even after more than 5000 bending cycles.^[^ ^66^ ^]^ However, the low stretchability and inherent yellow hue of PI restrict its use in applications that require significant flexibility or transparency. Despite these limitations, PI continues to be a preferred substrate in flexible electronics where durability is prioritized over extreme stretchability.^[^ ^20^ ^]^

In contrast, *PET substrates* have gained popularity in flexible and transparent electronics due to their high optical transmittance (>85%), low cost, and good chemical resistance.^[^ ^67^ ^]^ PET is particularly favored in transparent gas sensors, offering the flexibility needed for various wearable applications while maintaining transparency in the visible wavelength range. PET‐based sensors have shown excellent mechanical stability, withstanding 5000 bending cycles with a bending radius as small as 0.95 cm.^[^ ^68^, ^69^ ^]^ However, due to its relatively high tensile strength (2–4 GPa) and limited thermal stability, PET is not suitable for stretchable platforms or applications that involve high‐temperature processing (>100 °C).^[^ ^16^ ^]^ As a result, while PET is well‐suited for flexible applications, it is not an ideal candidate for stretchable or high‐temperature environments.

#### 2.1.2 Paper‐Based Substrates

Paper‐based substrates are also used in the fabrication of flexible and wearable gas sensors due to their flexibility, foldability, and low cost. These substrates are lightweight, recyclable, biocompatible, and environmentally friendly, offering the advantage of reducing electronic waste.^[^ ^17^, ^59^, ^67^, ^70^, ^71^ ^]^ Their self‐degradable nature further supports the shift toward sustainable technologies. For instance, Mirica et al.^[^ ^72^ ^]^ developed single‐walled carbon nanotube (SWCNT)‐based gas sensors on paper using a simple pencil drawing method. In Figure 1c, Quan et al. developed a completely flexible paper‐based gas sensor, incorporating a Ti_3_C_2_Tx‐MXene nonmetallic electrode and a Ti_3_C_2_Tx/WS_2_ gas sensing film. This design established both Ohmic contact and a Schottky heterojunction within a single gas sensing channel.^[^ ^73^ ^]^ However, paper substrates, due to their surface roughness and porosity, are not suitable for high‐quality thin‐film deposition.^[^ ^70^ ^]^ Despite their versatility, paper substrates are unsuitable for detecting VOCs because their hygroscopic nature can cause interference from absorbed humidity.^[^ ^70^ ^]^ Nonetheless, paper‐based sensors have been effectively used in moisture detection applications, such as monitoring respiration rates.^[^ ^59^, ^71^ ^]^ Additionally, tattoo papers have been explored for on‐body gas detection, offering a flexible and easy‐to‐apply platform for hazardous gas monitoring.^[^ ^57^, ^74^ ^]^

#### 2.1.3 Textile‐Based Substrates

Textiles are a promising alternative substrate for “in‐clothing” gas sensors due to their durability, breathability, flexibility, and washability.^[^ ^58^, ^67^, ^75^ ^]^ Textiles, made from animal (wool), plant (cotton), or synthetic (nylon) sources, possess various chemical and physical properties that make them suitable for wearable sensor applications.^[^ ^5^, ^29^, ^67^, ^76^ ^]^ As skin‐compatible materials, textiles have been considered for integrating gas sensors.^[^ ^5^, ^29^ ^]^

Gas sensors on textiles are either fabricated directly onto the fabric or integrated with an external sensor. In the direct method, textiles act as both the substrate and the sensing layer. For instance, Smith and Mirica^[^ ^58^ ^]^ developed multifunctional E‐textiles by modifying cotton and polyester fabrics with conductive 2D MOFs using a self‐assembly process. In the integration approach, external sensors are attached to textile substrates. Li et al.^[^ ^62^, ^77^ ^]^ functionalized cotton, leaf of the leaving plant, silk, and elastic threads and incorporated them into cotton fabrics for E‐textile applications (Figure 1d). Wu et al.^[^ ^75^ ^]^ demonstrated smart wearable devices by embedding NF‐structured single yarn sensors using techniques like knitting, braiding, and embroidering. Additionally, wrapping cotton and nylon yarns with sensing materials, and modifying electrospun fibers with metal oxides, significantly improved sensor performance.^[^ ^78^, ^79^, ^80^, ^81^ ^]^

#### 2.1.4 Other Substrates

For “on‐body” wearable gas sensors, the substrate must be flexible, conformable, and comfortable for the user. Conventional substrates like PET and PI lack the stretchability required for epidermal electronics, leading to instability during movement.^[^ ^56^ ^]^ Additionally, paper and textile substrates are unsuitable for depositing high‐quality sensing materials and fabricating dense circuits needed for advanced wearable electronics. Therefore, engineering stretchable substrates is essential for enhancing the stability and durability of these sensors.^[^ ^82^, ^83^ ^]^

Recent developments focus on structurally engineered stretchable substrates. For example, Lee et al.^[^ ^84^ ^]^ designed a “mogul”‐patterned PDMS substrate that efficiently absorbs stress, ensuring stable sensor performance. Similarly, they developed a skin‐inspired substrate with multi‐NF networks, where elastic polyurethane NFs were embedded between stiff P(VDF‐TrFE) layers in PDMS, mimicking skin‐like mechanical properties.^[^ ^85^ ^]^ Both approaches demonstrated stable sensing performance under strain, making them suitable for epidermal applications.^[^ ^84^, ^85^ ^]^

### 2.2 Design of Stretchable/Wearable Gas Sensors

Flexible electronics consist of circuits and components that retain functionality while being bent, though flexibility does not always equate to stretchability. Stretchable electronics, on the other hand, can be elongated and are more durable, making them closer to human skin in terms of mechanical properties.^[^ ^4^, ^86^ ^]^ Achieving flexibility can be done in two ways: reducing the thickness of materials or positioning active layers at the neutral mechanical plane. For instance, a wearable ECG system with an ultrathin structure (<3 μm) demonstrated reliable performance under cyclic bending, while a memory module placed at the neutral plane experienced less than 0.1% strain during bending.^[^ ^4^, ^87^ ^]^

Stretchable electronics can be achieved using two strategies: stretchable materials and structures. High‐aspect‐ratio nanomaterials like metallic nanowires are often arranged in percolation networks to accommodate strain.^[^ ^88^ ^]^ These nanomaterials can be processed on elastic substrates, such as PDMS or textiles.^[^ ^89^ ^]^ Nanomaterials can also be embedded beneath elastomer surfaces, forming nanocomposites for added stretchability.^[^ ^87^, ^90^ ^]^ For example, silver nanowire (AgNW)/PDMS nanocomposites are both highly conductive and stretchable.^[^ ^4^ ^]^ Additionally, blending nanomaterials into elastomers like PDMS can create flexible conductors, such as Ag–Au NWs dispersed in elastomers for stretchable electronics.^[^ ^91^ ^]^

Nonstretchable materials can be configured into stretchable structures using designs like waves, wrinkles, and serpentines (Figure 1i),^[^ ^86^, ^92^ ^]^ or more complex configurations such as fractals,^[^ ^93^ ^]^ origami, and kirigami.^[^ ^94^, ^95^ ^]^ Kirigami, for example, introduces cuts to impart stretchability. Takei et al. demonstrated a kirigami‐based wearable patch with multiple sensors, transforming a flexible PET substrate into a stretchable system.^[^ ^95^ ^]^

### 2.3 Electrodes for Wearable Gas Sensing Devices

Electrodes in wearable gas sensing devices are critical components that enable electrical functionality by connecting functional layers to the user interface. High conductivity is essential for these electrodes, and materials are often selected based on their ability to carry charges, regardless of their flexibility or stretchability.^[^ ^5^ ^]^ However, recent research has focused on balancing conductivity with flexibility using both material‐based and geometric engineering‐based approaches.^[^ ^18^, ^96^, ^97^ ^]^

A wide variety of nanomaterials are used for interconnects in wearable gas sensors, including transition metals, carbon allotropes, conducting polymers, and nanocomposites. Transition metal‐based nanomaterials are particularly popular due to their high electrical conductivity and ease of fabrication. Gold (Au) nanostructures are commonly used as electrode materials for wearable gas sensors despite their high cost, thanks to their excellent biocompatibility and resistance to oxidation (Figure 1i).^[^ ^68^, ^70^, ^98^, ^99^, ^100^, ^101^ ^]^ Other metals, such as platinum (Pt),^[^ ^80^, ^102^ ^]^ aluminum (Al),^[^ ^13^, ^103^ ^]^ silver (Ag),^[^ ^5^, ^58^, ^104^ ^]^ nickel (Ni),^[^ ^105^ ^]^ and copper (Cu),^[^ ^22^, ^26^, ^106^ ^]^ have also been widely used in electrode fabrication.

In addition to single‐metal electrodes, multimetal combinations have been explored to create multilayered electrodes with varying thicknesses, such as Cr/Au,^[^ ^47^, ^67^, ^85^ ^]^ Ti/Al/Ni/Au/Pt,^[^ ^84^ ^]^ Ni/Au,^[^ ^107^ ^]^ and Ag/Pd.^[^ ^108^ ^]^ Liquid metals, such as Galinstan (a mix of gallium, indium, and tin), have also been employed as interconnects due to their capacity for multiaxial deformation and excellent conductivity (Figure 1g).^[^ ^109^, ^110^ ^]^

Carbon‐based nanostructures have gained attention as alternative electrode materials due to their lower cost, despite being less conductive than metals. Various carbon allotropes have been used in wearable carbon‐based energy storage systems (WCESs), including 0D carbon black,^[^ ^20^, ^65^, ^82^, ^83^, ^111^ ^]^ 1D SWCNTs,^[^ ^65^ ^]^ and multiwall carbon nanotubes,^[^ ^25^, ^112^ ^]^ 2D graphene^[^ ^45^, ^50^, ^80^, ^113^, ^114^ ^]^ and reduced GO (rGO),^[^ ^115^, ^116^ ^]^ and 3D graphite ink.^[^ ^66^ ^]^

Conductive polymers are another option for electrode materials, valued for their tunable electrical conductivity, which can range from ≈1 × 10^−^^9^ to ≈1 × 10^5^ S cm^−^^1^.^[^ ^117^ ^]^ For instance, polyaniline (PANI) sulfonate (PSS)^[^ ^118^ ^]^ and polyester films^[^ ^103^ ^]^ have been applied as electrode materials.

To overcome the limitations of single‐material electrodes, nanocomposites combining different materials have been increasingly used in recent WCESs. Examples include PEDOT/PU films,^[^ ^119^ ^]^ AgNW–graphene composites,^[^ ^120^ ^]^ carbon/Prussian blue (PB) composites,^[^ ^111^ ^]^ Ag/AgCl,^[^ ^20^, ^66^, ^80^, ^82^, ^83^ ^]^ carbon paste‐Ag/AgCl,^[^ ^121^ ^]^ and Ag/AgCl/Ecoflex.^[^ ^112^ ^]^ These combinations offer enhanced conductivity and mechanical performance, making them ideal for wearable sensor applications (Figure 1e,f,h).

### 2.4 Gas Sensing Mechanism and Performance Evaluation of 2D Material‐Based Wearable Gas Sensors

In wearable gas sensors, the sensing mechanism predominantly relies on charge transfer rather than the surface‐adsorbed oxygen ion mechanism, as these sensors are designed to operate at RT without the need for integrated heaters. This is crucial for wearable applications, where maintaining low power consumption and ensuring user comfort are paramount.^[^ ^46^, ^122^ ^]^

In the charge transfer mechanism, gas molecules interact directly with the surface of the sensing material, causing the transfer of charge between the gas and the material. This interaction leads to changes in the material's electronic properties, such as conductivity or resistance.^[^ ^123^, ^124^ ^]^ For instance, in n‐type semiconductors, exposure to oxidizing gases like NO_2_ can cause electron withdrawal from the material, increasing the electron depletion layer (EDL) and resistance. Conversely, reducing gases like NH_3_ donate electrons, decreasing the EDL and resistance.^[^ ^125^ ^]^

Unlike the surface‐adsorbed oxygen ion mechanism, which relies on elevated temperatures to activate oxygen ion formation (e.g., O_2_^−^, O^−^, and O^2−^ at different temperature ranges), the charge transfer mechanism is inherently efficient at RT. This makes it ideal for wearable sensors, where integrating heaters is impractical due to energy and design constraints. Materials such as 2D semiconductors (e.g., graphene, TMDs, and MXenes) are particularly well‐suited for this mechanism due to their high surface area, tunable electronic properties, and excellent sensitivity to gas adsorption at RT. This approach not only enhances the practicality of wearable sensors but also broadens their application in real‐time environmental monitoring and healthcare diagnostics.^[^ ^123^, ^124^ ^]^

**Figure 2:**
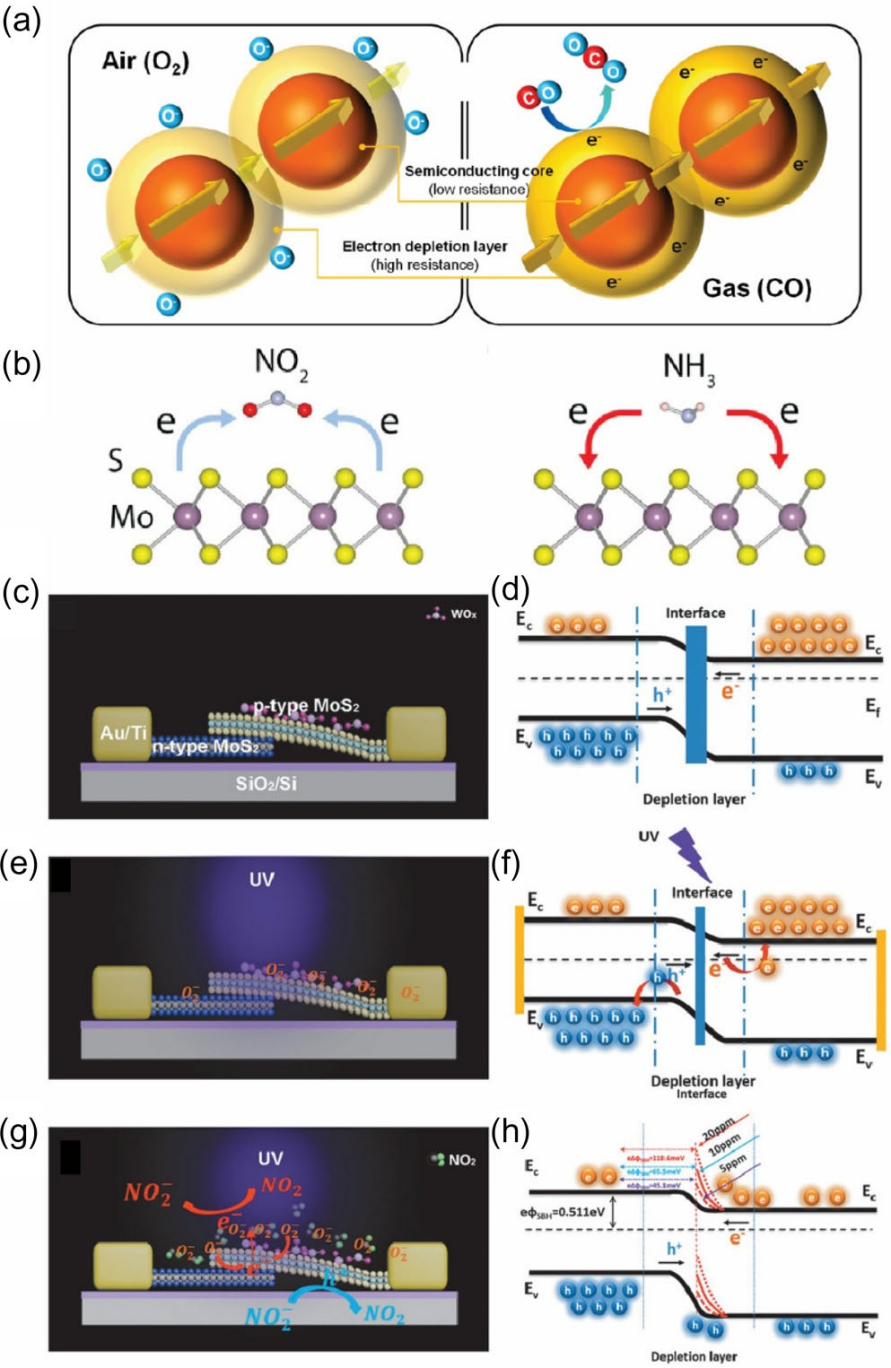
a) Gas sensing mechanisms in 2D material‐based gas sensors for n‐type semiconductors: oxidizing gases increase the EDL in n‐type materials, leading to higher resistance. Reproduced with permission.^[^ ^228^ ^]^ Copyright 2014, Elsevier. b) Schematic illustrations of the charge density differences in MoS_2_ upon interaction with gas molecules show that NO_2_ acts as an electron acceptor, withdrawing electrons from the MoS_2_ surface, while NH_3_ behaves as an electron donor, contributing electrons to the material. Reproduced with permission.^[^ ^129^ ^]^ Copyright 2015, Springer Nature. c,d) Schematic illustrations and corresponding energy band diagrams of a MoS_2_ p‐n junction: structure and band alignment in the dark, e,f) under UV irradiation showing enhanced charge separation, and g,h) under UV irradiation with NO_2_ exposure at varying concentrations (5, 10, and 20 ppm), demonstrating increased SBH due to electron‐withdrawing NO_2_ molecules. These diagrams highlight the influence of UV activation and gas adsorption on the electronic properties of the junction, supporting its application in sensitive NO_2_ gas sensing.^[^ ^229^ ^]^ Copyright 2020, John Wiley and Sons.

Noble metal decoration further enhances the gas sensing capabilities of 2D materials. Noble metals like gold (Au), silver (Ag), and palladium (Pd) have a higher work function than most n‐type sensing materials. When these metals come into contact with the sensing material, electrons are transferred from the sensing material to the noble metal, forming an EDL and Schottky barriers at the contact points.^[^ ^126^ ^]^ Exposure to oxidizing gases like NO_2_ increases the Schottky barrier height (SBH) and expands the EDL, modulating the sensor's resistance. Additionally, the electronic sensitization effect of noble metals plays a role, particularly when reducing gases like hydrogen (H_2_) interact with oxygen species on the noble metal surface, leading to electron transfer and significant resistance changes.^[^ ^127^, ^128^ ^]^ For n‐type sensing materials, the EDL forms on the surface, while for p‐type sensing materials, a hole accumulation layer (HAL) develops (**Figure** **2**a). This results in higher electrical resistance for n‐type materials and lower resistance for p‐type materials under vacuum conditions. Upon exposure to reducing gases, these gases react with the pre‐adsorbed oxygen species on the sensor's surface, releasing electrons back to the sensing layer. Consequently, the EDL and HAL layers shrink. For n‐type sensors, this leads to a decrease in electrical resistance, whereas for p‐type sensors, the resistance increases in the presence of reducing gases.^[^ ^3^ ^]^

Unlike the sensing mechanisms of conventional materials, where adsorbed oxygen ions play a critical role, many emerging 2D materials, such as graphene, TMDs, and MXenes, enable gas detection without requiring these oxygen ions. Instead, target gas molecules interact directly with the surface of these sensing materials through physisorption, facilitated by van der Waals forces or donor–acceptor electronic interactions. This interaction leads to a direct charge transfer mechanism, as depicted in Figure 2b. This charge transfer alters the carrier concentration in the sensing material, which, in turn, modifies its electrical resistance.

Oxidizing gases like NO_2_ extract electrons from the sensing material, while reducing gases such as NH_3_ donate electrons, leading to distinct changes in resistance. This direct charge transfer mechanism has been substantiated through extensive theoretical and experimental studies, including in situ photoluminescence (PL) investigations.^[^ ^129^ ^]^

Additionally, gas exposure can modulate the SBH at the interface between the sensing material and the metal electrode. The Schottky barrier arises from the difference in the work function of the metal and the electron affinity of the material. This modulation further enhances the sensing response by affecting the charge carrier dynamics at the interface.^[^ ^130^ ^]^

Composite material fabrication is another effective strategy for improving sensor performance. By combining two different materials, heterojunctions (p–n, n–n, or p–p) form at the interface. Figure 2c,h illustrates the schematic structure and band alignment of a MoS_2_ p–n junction under different environmental conditions. Under UV illumination, photogenerated carriers in the junction enhance charge separation, facilitating gas detection. When exposed to NO_2_ gas under UV light, the energy band diagram reveals a progressive increase in SBH with rising NO_2_ concentrations (5, 10, and 20 ppm), indicating electron withdrawal by NO_2_ molecules. This configuration demonstrates the potential of UV‐activated MoS_2_ p–n junctions for highly sensitive and selective NO_2_ detection at RT. Similar effects occur in n–n and p–p heterojunctions, where electron accumulation and depletion layers modulate the sensor's resistance during gas exposure.^[^ ^8^, ^131^ ^]^

To validate these mechanisms, researchers have employed both *theoretical calculations and in situ experiments*. *Density functional theory (* *DFT* *) simulations* provide insights into binding energies and charge transfer for gas molecules on 2D material surfaces. For instance, DFT has shown that a single NO_2_ can withdraw ≈0.1e^−^ from a pristine graphene sheet (resulting in hole doping), but if the graphene has a vacancy or is doped with N, the charge transfer can double, correlating with increased sensitivity. Likewise, DFT on MoS_2_ predicted that introducing S‐vacancies lowers the adsorption energy for NH_3_ from −0.2 to −0.5 eV, meaning that NH_3_ binds more strongly—consistent with experimental observations that defective MoS_2_ is more sensitive to NH_3_.^[^ ^132^ ^]^ On the experimental side, in situ X‐ray photoelectron Spectroscopy (XPS) *and Raman* have been used: for example, in situ XPS on a SnS_2_ sensor showed a higher fraction of Sn(II) species when exposed to H_2_S, directly evidencing a surface redox reaction.^[^ ^133^ ^]^ In situ Raman spectroscopy on GO revealed that the D/G intensity ratio increases upon NO_2_ exposure (indicating charge transfer‐induced changes in GO's structure) and reverts after purging NO_2_, supporting a reversible physisorption mechanism.^[^ ^134^ ^]^

*Performance evaluation* of gas sensors typically involves measuring the *sensitivity (response)*, *response and recovery times*, *limit of detection (* *LOD*), *selectivity*, and *stability*.^[^ ^135^ ^]^ The sensor response refers to the change in the measured electrical signal (e.g., current, resistance, or voltage) when the sensor is exposed to analytes. For chemiresistive sensors, the response is often defined as a fractional change in resistance: $\text{Response}\left(R\right)=\frac{R_{\text{gas}}-R_{\text{air}}}{R_{\text{air}}}\times 100\%$ for reducing gases (which increase resistance in n‐types) or the inverse for oxidizing depending on convention. In this review, the response is consistently reported as a percentage increase in baseline resistance for n‐type materials or a percentage decrease for p‐type materials, unless otherwise specified. The response time is defined as the time required to reach 90% of the full response after the introduction of the target gas, while the recovery time is defined similarly upon the removal of the gas. Table 3 and 4 in Section 3 summarize these metrics for a variety of flexible sensors.

The goals for wearable sensors are often *room‐temperature operation* (to minimize power), *rapid response (seconds)*, *low* *LOD* *(ppm to ppb)*, and *good selectivity* to the gas of interest. Achieving all simultaneously is challenging. 2D materials have achieved remarkable room‐temperature sensitivity for example, a few ppb NO_2_ with BP^[^ ^136^ ^]^ and sub‐ppm NH_3_ with MXenes.^[^ ^137^, ^138^ ^]^ Response times at RT can be somewhat longer (tens of seconds to minutes) if no extra activation (like UV illumination or heating) is used, due to the kinetics of adsorption/desorption. Some strategies to improve kinetics include *built‐in microheaters* (self‐heating elements to slightly elevate temperature locally) or *UV* *light emitting diode (* *LED* *) illumination* to help desorb molecules and accelerate surface reactions.^[^ ^39^, ^139^ ^]^ Power consumption remains a concern, but many 2D sensors can operate with only a few volts bias at microamp currents (submilliwatt power), and innovations like self‐heating WS_2_ sensors utilize the sensing current itself to generate a small temperature rise (≈50 °C) to speed up response.^[^ ^140^ ^]^

Selectivity can be improved by material choice (as different gases interact differently with each material) and by sensor array approaches (pattern recognition). For instance, MXene sensors are highly selective to NH_3_ over acetone and ethanol, while MOF‐coated sensors can be tailored to single gases like formaldehyde amidst others.^[^ ^137^, ^138^ ^]^

The signal‐to‐noise ratio (SNR) quantifies the ratio between the desired signal and background noise, with a high SNR being especially important for wearable sensors, where mechanical deformations may degrade signal quality.

Reproducibility measures the sensor's ability to deliver consistent results under varying conditions, ensuring reliability across multiple uses. Stability reflects the sensor's capacity to maintain consistent performance over time under the same conditions. Finally, the dynamic range defines the sensor's operational span, from its LOD to its upper detection limit, determining the breadth of its application.^[^ ^5^, ^135^ ^]^

The combination of experimental and theoretical work is advancing the understanding of these mechanisms, guiding the design of more sensitive and selective sensors.

### 2.5 Fabrication and Integration Technique of Wearable Gas Sensor

To realize flexible gas sensors based on 2D materials, suitable fabrication techniques are required to deposit or pattern the sensing materials and electrodes onto flexible substrates. Key considerations include low processing temperatures (to accommodate plastic substrates), scalability for large‐area or batch production, and maintaining the intrinsic properties of the 2D materials. This review outlines common fabrication approaches to address these requirements.

#### 2.5.1 Solution Processing and Coating

**Figure 3:**
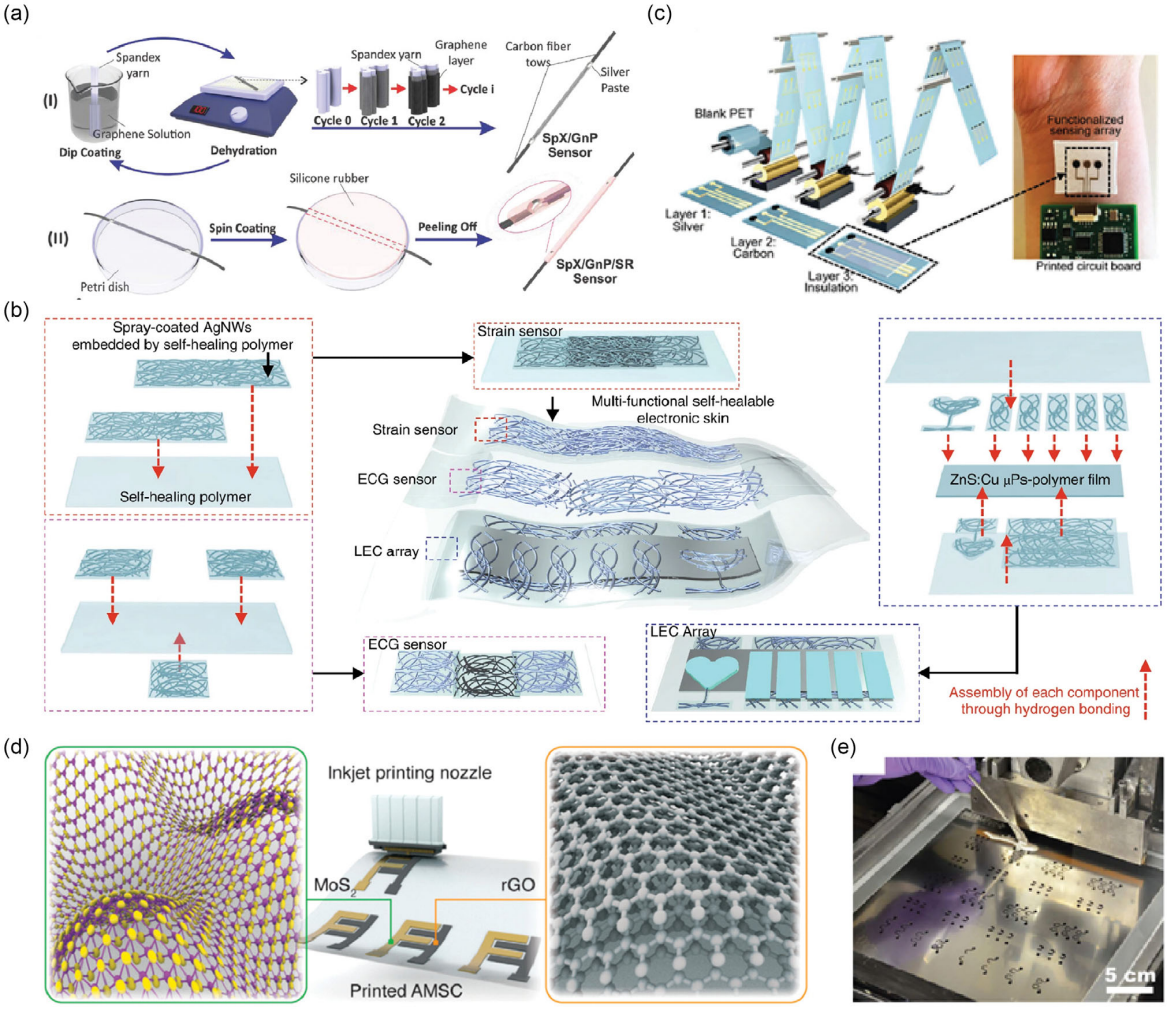
a) Illustration of the sensor fabrication steps using solution process technique (dip coating). Adapted with permission from.^[^ ^230^ ^]^ Copyright, 2017, Wiley‐VCH. b) Spray‐coating manufacturing technique used to develop a wearable system integrating a strain sensor, ECG sensor, and light‐emitting capacitor (LEC) array. Adapted with permission from.^[^ ^87^ ^]^ Copyright 2018, Springer Nature. c) R2R printing technique applied for the production of electrochemical sensors. Adapted with permission from.^[^ ^148^ ^]^ Copyright 2018, American Chemical Society. d) Screen‐printing process for fabricating electrochemical sensors based on CNTs. Adapted with permission from.^[^ ^146^ ^]^ Copyright 2015, American Chemical Society. e) Schematic representation of an inkjet‐printed asymmetric microsupercapacitor (AMSC), featuring 3D 1T‐phase chemically exfoliated MoS_2_ (left) and rGO (right). Reproduced with permission.^[^ ^231^ ^]^ Copyright 2020, American Chemical Society.

Many 2D materials (graphene, GO, MXenes, and some MOF nanosheets) can be dispersed in solvents and thus deposited by solution techniques. Deep coating, *d* *rop‐casting*, and *spin‐coating* are simple methods to coat a substrate with a thin film of 2D material (**Figure** **3**a). For example, a dispersion of chemically exfoliated MoS_2_ or MXene can be dropped onto a PI film and dried, forming the sensing film.^[^ ^141^ ^]^ However, these methods can lack uniformity control. *Vacuum filtration* through a membrane is a great way to assemble a uniform film: One can filter a MXene or graphene solution and then transfer the resulting film (like a paper) onto a flexible substrate.^[^ ^126^ ^]^ Ghosh et al.^[^ ^7^ ^]^ used vacuum filtration to deposit a uniform rGO film on paper for NH_3_ sensing. *Spray‐coating* (Figure 3b) is another scalable approach, where an ultrasonic sprayer deposits layers of material; Chen et al.^[^ ^142^ ^]^ spray‐coated rGO onto textile for a foldable H_2_ sensor. These solution methods are simple and can cover large areas, but often require multiple steps to define patterns (e.g., using shadow masks for spray or spin‐coating areas).

#### 2.5.2 Printing Techniques (Inkjet, Screen, and 3D Printing)

Printing is an attractive route for scalable and cost effective fabrication of wearable sensors. This techniques are capable of producing large‐area sensor patterns on flexible substrates.^[^ ^143^, ^144^ ^]^ *Inkjet printing* (Figure 3e) involves formulating an ink (2D material + solvent + stabilizers) and digitally patterning it. Spieser et al.^[^ ^143^ ^]^ printed graphene and MXene‐based inks onto polymer substrates, achieving resolution ≈50 μm. Inkjet is additive and mask‐less but requires optimized rheology of inks and can be slow for large areas. *Screen printing*, on the other hand, uses a physical mesh stencil to print patterns of paste. It's rapid and industrially proven (like in printed electronics).^[^ ^145^ ^]^ Bandodkar et al.^[^ ^146^ ^]^ screen‐printed a CNT/graphene composite on PDMS to serve as stretchable electrodes in a sensor array (Figure 3d). Figure 3c presents *3D printing* (e.g., direct ink writing), which has also been used to create sensor structures with novel architectures (like a 3D lattice of graphene).^[^ ^147^ ^]^ A challenge with printing pure 2D materials is maintaining conductivity at high ink loading; often binders are added, which must be chosen to not insulate the material. Overall, printing offers a pathway to high‐volume manufacturing of sensor elements at low cost, and roll‐to‐roll (R2R) printing of conductive patterns is already feasible.^[^ ^148^ ^]^

#### 2.5.3 CVD and Transfer

For high‐quality 2D crystals like monolayer graphene or TMDs, chemical vapor deposition (CVD) growth on metals (for graphene) or insulating substrates is common. To use these in flexible sensors, a transfer step to a polymer substrate is needed (e.g., using poly methyl methacrylate‐assisted transfer for graphene from Cu foil to PET). CVD‐grown graphene and MoS_2_ have been integrated into flexible FET gas sensors with excellent uniformity and performance.^[^ ^11^ ^]^ While CVD yields great material quality, it is typically limited to smaller areas or requires expensive equipment, and transferring without cracks or contamination is nontrivial. Novel techniques like directly growing graphene on PI by laser carbonization avoid transfer altogether. Laser‐induced graphene (LIG) uses a CO_2_ laser to convert the surface of PI into a porous graphene foam and can pattern electrodes and sensors in one step—for instance, a breathable NO_2_ sensor was made this way on Kevlar fabric.^[^ ^149^ ^]^

#### 2.5.4 Layer‐by‐layer (LbL) Assembly and Self‐Assembly

For constructing multilayer structures (like alternating layers of GO and polymer, or MXene and other 2D sheets), LbL assembly via electrostatic attraction can be used. This can create ultrathin films with nanometer control. Although more common in sensing films for strain or bio‐sensors, one could envision LbL assembly to form, say, a GO/PANI multilayer sensitive to gases.^[^ ^150^ ^]^ *Self‐assembly* during solvent evaporation (like drop‐casting and letting coffee‐ring effects create patterns, or using templating) has also been employed to position 2D materials in particular layouts. For example, an evaporative assembly method was used to deposit MOF particles at specific locations on a graphene film for better selectivity.^[^ ^39^ ^]^

Given these options, the fabrication strategy often depends on the material form (solution‐processable or not) and the desired device geometry. To create a robust wearable sensor, *hybrid fabrication* is common: for example, screen print silver electrodes on PI, then drop‐cast a 2D material film bridging them, and then encapsulate. Or inkjet print both the 2D material and the contacts in successive steps (multimaterial printing). Ensuring alignment and repeatability is key for arrays or mass production.

In terms of *scalability*, printing and coating methods are most promising. Indeed, researchers have printed arrays of tens of graphene sensors on a flexible substrate in one go.^[^ ^131^ ^]^ As an example of high‐volume potential, a R2R gravure printing (Figure 3f) system recently demonstrated patterning of graphene sensor structures over meter lengths, with each sensor responding reliably to target gases.^[^ ^148^, ^151^ ^]^ This indicates that, beyond lab demonstrations, manufacturing methods are catching up to allow 2D material sensors to be produced in quantity.

#### 2.5.5 Post‐fabrication Integration

Flexible gas sensors often need to be integrated with a readout system or wireless transmitter for Internet of Things (IoT) use. Thus, flexible printing of conductive traces to a Bluetooth module or using flexible printed circuit boards (PCBs) to mount the sensor is another layer of fabrication. For now, many prototypes connect to rigid electronics via wire bonding or zero insertion force (ZIF) connectors, but fully flexible integrated systems (like a sensor on PI connected to a flexible near‐field communication tag antenna printed alongside it^[^ ^152^ ^]^) have been reported, showing the feasibility of completely wearable, wireless gas sensing patches.

In summary, a combination of low‐temperature deposition, innovative printing, and careful integration allows 2D material sensors to be realized on flexible and wearable platforms. Each fabrication method offers unique benefits, depending on the required film thickness, substrate compatibility, and production scale (**Table** **1**).

**Table 1:** Summary of 2D material‐based sensor fabrication methods.

Fabrication method	Scalability	Flexible substrate compatibility	Notes
Solution processing (drop‐casting, spin‐coating, deep coating)	Medium	High	Simple methods; less uniformity; requires patterning steps
Vacuum filtration + transfer	Medium	High	Uniform films; can be transferred onto flexible substrates; scalable for lab scale
Spray coating	High	High	Fast, scalable; needs masking for patterns; suitable for textiles
Inkjet printing	Medium	High	Digital, mask‐less; ink rheology critical; slower for large areas
Screen printing	High	High	Fast, industry‐ready; requires screen masks; good for batch production
3D printing (direct ink writing)	Medium	High	Enables complex 3D architectures; slower; ink formulation critical
CVD growth + transfer	Medium (wafer‐scale)	Medium (requires transfer step)	High quality films; moderate throughput; transfer can introduce defects
LIG	High	High (direct on PI)	One‐step direct patterning; porous graphene; highly compatible with flexible substrates
LbL assembly	Low	Medium	Precise nanometer‐thick multilayers; slower; niche applications
Self‐assembly (evaporation, templating)	Medium	Medium	Patterns formed naturally during evaporation; alignment less controlled
R2R printing	Very high	Very high	Industrial‐scale continuous production; proven for conductive patterns

## 3 2D Materials for Flexible Gas Sensors

The 2D materials exhibit remarkable properties that make them highly suitable for flexible gas sensor applications. Their atomic‐scale thickness and high surface‐to‐volume ratios enhance chemical reactivity and enable seamless integration into device fabrication processes. Typically ranging from atomic layers to a few microns in thickness and extending laterally up to several centimeters, these materials are ideal for large‐scale assembly without compromising their intrinsic properties.^[^ ^153^ ^]^

Among 2D materials, graphene stands out for its exceptional mechanical, thermal, chemical, and optoelectronic properties. Its atomic‐scale thickness facilitates incorporation into thin‐film fabrication via direct transfer techniques, making it compatible with flexible and wearable electronic devices. Graphene demonstrates high tensile strength, with a Young's modulus of 1000 GPa and a strain limit of about 25%, allowing it to endure significant deformation. Furthermore, its high optical transparency (>90%) and linear optical conductivity in the zero‐frequency electromagnetic field make it a preferred material for advanced sensor applications.^[^ ^146^ ^]^

The family of 2D materials has expanded significantly and now includes BP, TMDs, hBN, metal oxides (MOs), MOFs, covalent organic frameworks, rGO, nitride‐based nanomaterials, MXene compounds, and other 2D compounds.^[^ ^131^, ^154^, ^155^, ^156^ ^]^ These materials offer diverse properties, such as tunable conductivity, bandgap modulation, and high chemical reactivity, broadening their applicability in gas sensing. Advantages and disadvantages of various 2D materials for flexible gas sensors are summarized in **Table** **2**.

**Table 2:** Advantages and disadvantages of various 2D materials for flexible gas sensors.^[^ ^3^ ^]^

Material	Advantages	Disadvantages
TMDs	High mechanical flexibility, ease of exfoliation, tunable bandgaps	Long recovery time, low sensing response in pristine form
BPs	High surface area, tunable bandgap, good electrical conductivity, low sensing temperature, good flexibility	Low long‐term stability at RT, low sensing response in pristine form
2D nitride nanocomposite	Simple preparation, tunable electronic and morphological structure, good stability	Large bandgap, low electron mobility, low sensing response in pristine form
MXenes	High metallic conductivity, high surface area, tunable bandgap, tunable surface groups, low operating temperature, good flexibility, low toxicity	Easy agglomeration, low sensing response in pristine form
GO	High surface area, surface functional groups and defects, scalable synthesis, good flexibility	Very high resistance, low sensing response in pristine form
rGO	High electrical conductivity, abundant defects and functional groups, low sensing temperature, high surface area, good flexibility, scalable synthesis	Low sensing response in pristine form
Graphene	High electrical conductivity, high surface area, good mechanical flexibility, low sensing temperature	Ease of agglomeration, lack of bandgap, long response and recovery time, difficulty in large‐scale synthesis
MOFs	High surface area, abundant porosity, tunable framework and bandgap, low operating temperature, good flexibility	Moderate thermal stability, low sensing response in pristine form

The conductivity of 2D materials can range from insulating to metallic, depending on their bandgap. This property can be adjusted using methods such as doping, defect engineering, functionalization, or controlling the number of layers.^[^ ^157^ ^]^ For example, molybdenum disulfide (MoS_2_) monolayers exhibit tunable bandgap properties under mechanical force or an electric field, making them efficient for signal transduction in gas sensors. Additionally, their larger lateral size compared to 1D counterparts ensures better electrical contact, enhancing their integration into flexible and stretchable devices.^[^ ^158^ ^]^

The unique combination of high tensile strength, flexibility, and outstanding optical, physical, chemical, and thermal properties positions 2D materials as ideal candidates for flexible gas sensors. Their compatibility with thin‐film fabrication processes and their ability to maintain functionality under mechanical stress make them indispensable for next‐generation wearable sensing technologies.^[^ ^125^ ^]^

### 3.1 2D TMDs‐Based Flexible Gas Sensors

2D TMDs are highly promising materials for developing flexible and wearable gas sensors due to their tunable bandgaps, high electrical conductivity, mechanical flexibility, and stability. TMDs are represented by the formula MX_2_, where M is a transition metal such as molybdenum (Mo), tungsten (W), or zirconium (Zr) and X is a chalcogen such as sulfur (S), selenium (Se), or tellurium (Te).^[^ ^9^, ^159^ ^]^

These materials possess a high surface area due to their unique 2D morphology, which enhances their interaction with gas molecules. Their structure comprises covalent bonds within individual layers and weak van der Waals forces between layers, enabling easy exfoliation into single layers.^[^ ^160^ ^]^ This combination of properties makes TMDs ideal for applications requiring flexible and stable gas sensors with enhanced sensitivity and selectivity.

The exceptional mechanical and electrical properties of MoS_2_ have driven its extensive application in flexible and wearable electronic devices. Zhao et al.^[^ ^161^ ^]^ developed a highly sensitive and flexible MoS_2_‐based gas sensor capable of operating at low temperatures. The 2D MoS_2_ material was directly synthesized on a flexible PI substrate using a low‐temperature CVD process at 200 °C. This innovative fabrication method enabled the production of a gas sensor with excellent flexibility and robust performance.

**Figure 4:**
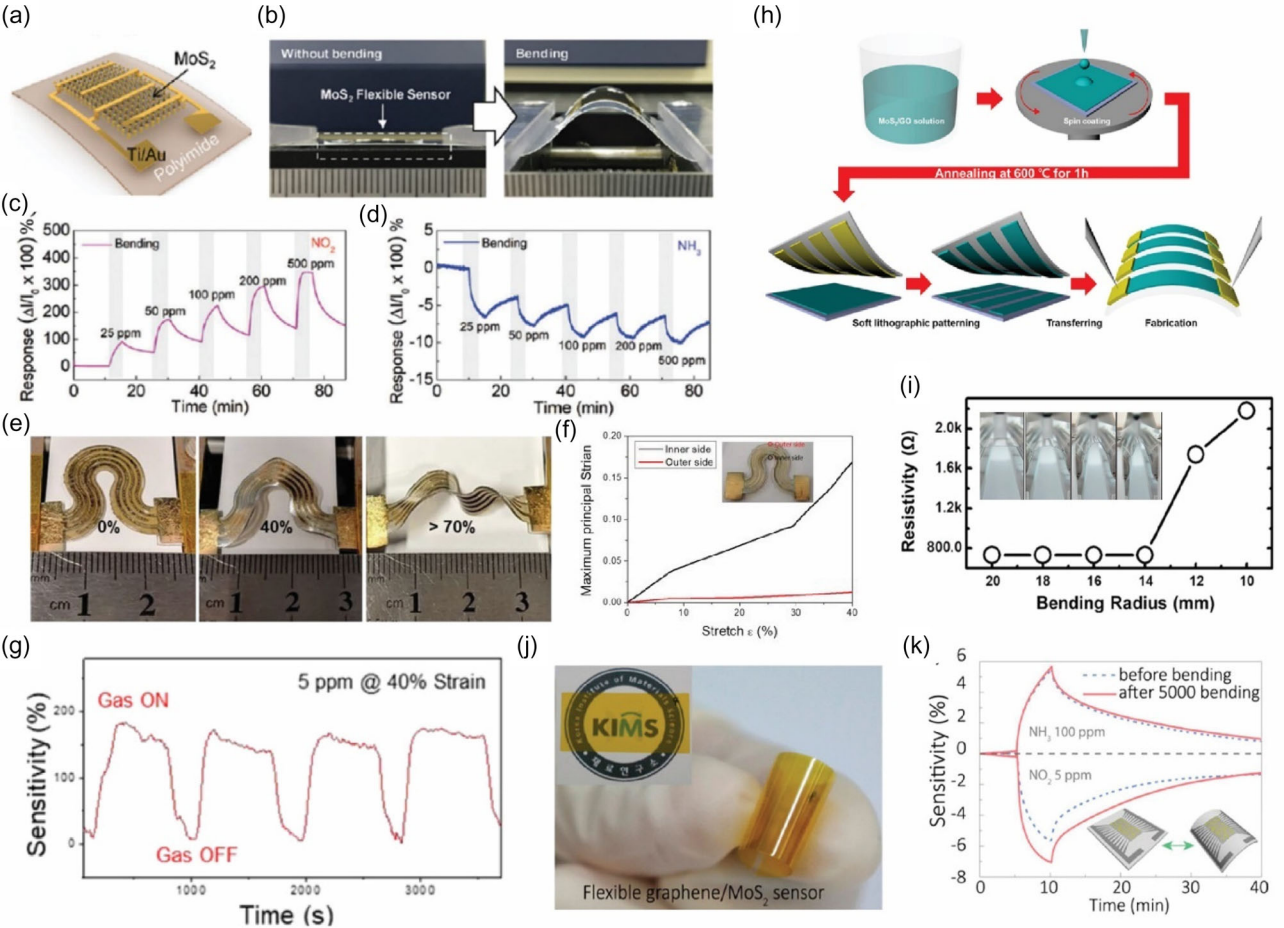
a) Schematic illustration and b) photograph of a flexible MoS_2_ gas sensor, demonstrating its structure and performance under bending and nonbending conditions. c,d) Gas sensing response to NO_2_ and NH_3_ at varying concentrations under both bending and flat conditions, highlighting the sensor's robustness. Reproduced with permission.^[^ ^161^ ^]^ Copyright 2018, The Royal Society of Chemistry. e) Photograph of lateral stretching in VA‐2D MoS_2_ layers. f) Plot correlating stretch rate with principal strain at specific locations shown in the inset. g) Sensor sensitivity under 5 ppm NO_2_ exposure, demonstrating consistent performance under 40% strain. Reproduced with permission.^[^ ^162^ ^]^ Copyright 2020, American Chemical Society. h) Schematic representation of the MoS_2_/rGO sensor. i) Sensor resistivity under bending conditions, with inset images showing the bent device on PET. Adapted with permission.^[^ ^163^ ^]^ Copyright 2018, Elsevier. j) Photograph of a graphene/MoS_2_ sensor on a PI substrate and a semitransparent device printed on paper, featuring the KIMS logo (inset). k) Comparison of sensor sensitivity before and after bending cycle tests, with a 3D schematic inset illustrating the bending test conditions. Reproduced with permission.^[^ ^66^ ^]^ Copyright 2015, American Chemical Society.

**Figure** **4**a–d illustrates the fabricated MoS_2_ sensor and the corresponding bending test setup. The device's flexibility was demonstrated through a bending test conducted over 6000 cycles. The sensor's gas response to NO_2_ and NH_3_ remained consistent before and after bending, showcasing its structural integrity and reliable performance under mechanical stress. This durability underscores the sensor's potential for wearable and flexible gas detection applications.

The vertically aligned (VA) layers in MoS_2_ exhibit a significant density of surface‐exposed edge sites due to their intrinsic crystallographic anisotropy, which results in numerous dangling bonds. These features make VA‐MoS_2_ highly effective for gas sensing applications. In this context, Islam et al.^[^ ^162^ ^]^ reported the fabrication of VA‐MoS_2_ nanosheets on mixed polymeric patterns for detecting NO_2_ gas. The integration of serpentine‐patterned VA‐2D MoS_2_ layers with sequential lateral stretching of Au electrodes is shown in Figure 4e. As the strain ratio increases, the sample undergoes out‐of‐plane orientation distortion, becoming particularly pronounced at strains exceeding 70%.

The gas sensor demonstrated a sensitivity of ≈200% at 5 ppm NO_2_, with no significant degradation in performance even under 40% strain, as illustrated in Figure 4g. This stability under strain highlights the sensor's robustness and suitability for practical applications. The serpentine pattern effectively minimized the distortion of the VA‐MoS_2_ layers’ alignment, preserving their surface‐exposed dangling bonds, which are crucial for gas sensing performance.

Moreover, the maximum principal strain was concentrated at the inner edges of the serpentine pattern with high curvature, as shown in Figure 4f. This strain concentration became more pronounced with increasing stretching, as evidenced by the stretch rate versus maximum principal strain plots. These findings underscore the innovative design and mechanical resilience of the sensor, ensuring its functionality under various deformation conditions. Combined with its sensitivity and durability, this sensor design shows significant potential for real‐world flexible gas sensing applications.^[^ ^162^ ^]^

The combination of MoS_2_ with carbon‐based materials like rGO takes advantage of their high conductivity and flexibility, making these composites highly effective for gas sensing applications. Jung et al.^[^ ^163^ ^]^ reported a flexible gas sensor was fabricated on a PET substrate using a patterned MoS_2_/rGO composite, as shown in Figure 4h. This composite demonstrated a significant improvement in sensitivity, being at least four times more responsive than a sensor utilizing pure rGO alone, underscoring the synergistic benefits of the hybrid material.

The sensor also exhibited exceptional mechanical flexibility. As depicted in Figure 4i, its electrical resistance remained stable even after repeated bending to a radius of 14 mm, highlighting its durability and resilience under mechanical stress. This robust performance under deformation reinforces its potential for use in flexible and wearable gas sensing applications.

These advancements in MoS_2_/rGO composites complement the earlier discussed VA MoS_2_ sensors by enhancing sensitivity while maintaining mechanical robustness. Together, they illustrate the adaptability of MoS_2_‐based composites in developing next‐generation flexible and wearable gas sensing technologies.^[^ ^163^ ^]^

Similarly, Cho et al.^[^ ^66^ ^]^ investigated the gas‐sensing performance of a flexible sensor fabricated with a MoS_2_‐graphene composite on a PI substrate, as shown in Figure 4j. This sensor was tested for detecting NO_2_ and NH_3_, demonstrating robust performance under challenging conditions. Specifically, Figure 4k illustrates that the sensitivity of the graphene/MoS_2_ composite sensor remained stable even at elevated temperatures of 150 °C, with no significant degradation observed even after 5000 bending cycles.

The superior thermal stability of the PI substrate was critical in maintaining the sensor's functionality under such extreme conditions, making it an ideal choice for flexible and wearable device testing. This study highlights the compatibility of graphene/MoS_2_ composites with PI substrates, emphasizing their potential for applications requiring both thermal resilience and mechanical flexibility.

These findings further strengthen the case for hybrid 2D material‐based composites, building on previous discussions about MoS_2_/rGO composites. Together, these studies showcase the versatility and durability of 2D material hybrids for advanced gas‐sensing technologies in flexible and wearable formats.^[^ ^66^ ^]^

MoS_2_‐based composites have also been explored for detecting harmful gases. Yan et al.^[^ ^164^ ^]^ reported a flexible MoS_2_/PANI sensor capable of detecting NH_3_ with a response of 25 at 10 ppm. The sensor maintained performance under 30% strain and after 500 stretching cycles, attributed to the synergistic effects of the composite network, efficient charge transfer, and large surface area of 2D MoS_2_ layers.^[^ ^164^ ^]^

WS_2_ exhibits excellent potential for flexible gas sensors due to its mechanical strength, oxidation resistance, and adjustable bandgap. Hao et al.^[^ ^165^ ^]^ developed a WS_2_/Si thin‐film sensor incorporating a 3‐nm Pd layer using magnetron sputtering. This sensor demonstrated stable responses to H_2_ under bending with radii of 10 mm and 5 mm, and its sensitivity declined by only 7.3% after 50 bending cycles, showcasing its high flexibility and durability.^[^ ^165^ ^]^

**Figure 5:**
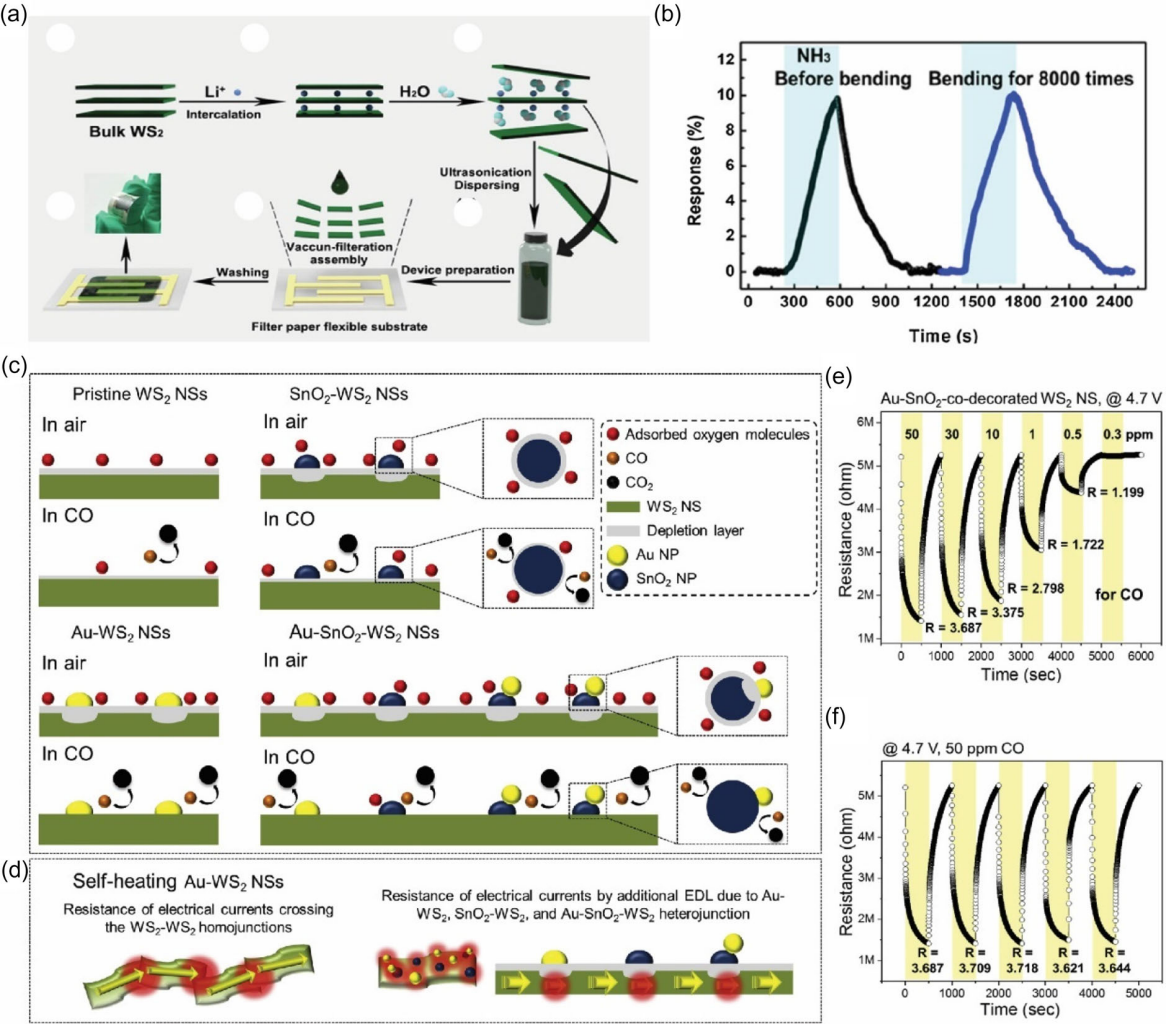
a) Schematic illustration of the synthesis and fabrication process of the Li‐WS_2_ gas sensor. b) Response curves of the sensor to 500 ppm NH_3_ before and after 8000 bending cycles. Reproduced with permission.^[^ ^166^ ^]^ Copyright 2022, Elsevier. c) Schematic representation of the CO sensing mechanism in bare WS_2_ NSs, SnO_2_‐decorated WS_2_ NSs, Au‐decorated WS_2_ NSs, and Au‐SnO_2_‐co‐decorated WS_2_ NSs. d) Schematic illustration of the self‐heating effect. e) Dynamic resistance curves of the Au‐SnO_2_‐co‐decorated WS_2_ NS sensor at various CO gas concentrations under a driving voltage of 4.7 V. f) Repeatability curve for 50ppm CO gas at 4.7 V. Reproduced with permission.^[^ ^167^ ^]^ Copyright 2021, Elsevier.

In a separate study, Qin et al.^[^ ^166^ ^]^ introduced Li intercalation into WS_2_ (Li‐WS_2_) on filter paper to create defects and improve NH_3_ sensing (**Figure** **5**a). Their flexible Li‐WS_2_/WS_2_ sensor achieved a remarkable ≈20% resistance drop for 500 ppm NH_3_ at RT and maintained that performance after *8000 bending cycles* (*10 mm radius*). The introduction of Li‐induced oxygen adsorption sites enhanced gas interaction, increasing sensitivity by ≈3× compared to pristine WS_2_, which in turn amplified the NH_3_ reaction a clear example where purposeful defect engineering and flexible substrate yielded high performance. Figure 5b confirmed negligible sensitivity change before versus after bending tests.

Further advancements were reported by Kim et al.^[^ ^167^ ^]^ who developed WS_2_‐SnO_2_ sensors co‐decorated with Au for room‐temperature CO sensing. The CO sensing mechanisms for pristine WS_2_ nanosheets (NSs) and their modifications with SnO_2_ and Au NPs are illustrated in Figure 5c. In pristine WS_2_ NS sensors, oxygen molecules adsorb on the surface, forming $O_{2}^{-}$ ions via $O_{2}\left(\text{ads}\right)+e^{-}\to O_{2}^{-}$, creating an EDL. Interaction with CO gas converts adsorbed oxygen to CO_2_, releasing electrons and reducing resistance.

For SnO_2_‐decorated WS_2_ NSs (Figure 5c), the higher sensitivity of SnO_2_ narrows the EDL, while electron transfer from WS_2_ to SnO_2_, driven by SnO_2_'s higher work function, enhances sensitivity. Au decoration (Figure 5c) further increases oxygen adsorption and accelerates electron transfer, while co‐decoration with Au and SnO_2_ (Figure 5c) synergistically modulates EDLs, maximizing response.

These sensors operate efficiently at RT due to self‐heating via Joule heating, where applied voltage generates heat within WS_2_ grains and interfaces, enhancing performance (Figure 5d).

Figure 5e shows the sensor's dynamic resistance to CO concentrations ranging from 50 to 0.3 ppm, demonstrating high sensitivity and the capability to detect low levels of CO gas. Repeatability tests (Figure 5f) confirm consistent responses over five cycles at 50 ppm CO, showcasing reliability and potential for real‐world applications. These findings, complementing NH_3_ sensing advancements, highlight WS_2_‐based sensors’ versatility in detecting diverse gases effectively.^[^ ^167^ ^]^

**Figure 6:**
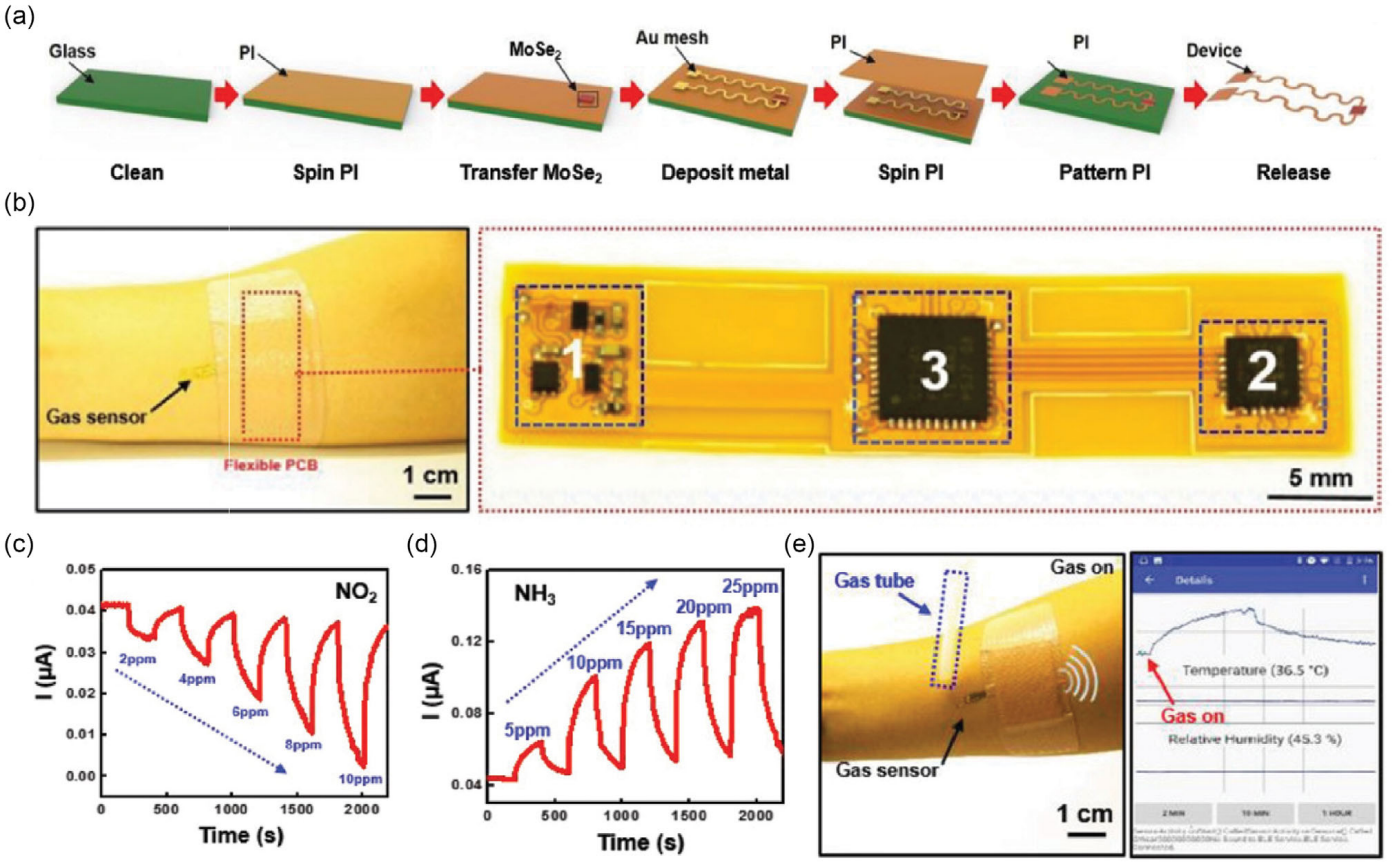
a) Experiment demonstrating NO_2_ and NH_3_ gas sensing properties on human skin. b) Optical image of the sensor assembly with flexible PCB under the bandage, highlighting key circuit components: (1) voltage divider, (2) amplifier, and (3) microcontroller. c) Response curve of the sensor to varying concentrations of NO_2_ at 5 V. d) Response curve of the sensor to varying concentrations of NH_3_ at 5 V. e) Gas response visualization on a smartphone application under active gas flow. Reproduced with permission.^[^ ^56^ ^]^ Copyright 2019, Wiley‐VCH.

MoSe_2_, another TMD with high charge carrier mobility and stability, has also been applied in flexible gas sensors. Guo et al.^[^ ^56^ ^]^ introduced a flexible and wearable gas sensor based on MoSe_2_, designed for the detection of both NH_3_ and NO_2_ gases. The fabrication process, which included steps such as preparing the PI substrate, transferring the MoSe_2_ layer, metallization, patterning, and final device release, is illustrated in **Figure** **6**a. The sensor's wearability was demonstrated by testing it directly on the skin under ambient conditions (RT) in the presence of NH_3_, as shown in Figure 6b. The experimental setup was operated in a controlled environment within a fume hood. To evaluate its real‐time performance, the sensor's current output was monitored via a mobile application. When the gas tube delivering NH_3_ was positioned near the sensor, the current trace on the app increased, confirming gas detection. Conversely, removing the tube caused the trace to naturally return to its baseline, as shown in Figure 6e. This single‐cycle test in a 2‐min interval validated the system's operational reliability. Additionally, the repeatability of the sensor was assessed through a 10‐min continuous pulsed gas response. The sensor successfully detected repeated gas pulses, demonstrating its ability to recover to baseline after each exposure, a key feature for practical applications. Figure 6c,d depicts the response curves of the sensor to various concentrations of NO_2_ and NH_3_ gases at an applied voltage of 5 V, further emphasizing its sensitivity and reliability. This MoSe_2_‐based gas sensor exemplifies the potential of flexible, wearable technologies for real‐time environmental and health monitoring, seamlessly integrating advanced materials and smartphone applications for practical use.^[^ ^56^ ^]^

WSe_2_, while less explored than WS_2_, has shown promise for flexible sensing applications. Cho et al.^[^ ^168^ ^]^ developed a NbSe_2_/WSe_2_ sensor on a PET substrate, achieving ≈30% response to NO_2_ and NH_3_ at 5 ppm and 100 ppm, respectively. The sensor retained performance after 10,000 bending cycles and was robust enough to function after being sewn onto clothing and washed.^[^ ^168^ ^]^

*SnS_2_* and *SnSe_2_* are TMDs of group IV elements that have also been explored. Huang et al.^[^ ^138^ ^]^ grew SnS_2_ nanoflakes on PI and detected NO_2_ with good sensitivity at RT, partly because SnS_2_ provides abundant sulfur vacancies as active sites. They observed a response of ≈40% at 1 ppm NO_2_, and importantly, the sensor kept working after repetitive flexing (up to 4% strain). SnS_2_'s stability in ambient (e.g., less prone to oxidation than BP) contributed to reliable operation.

Pyeon et al.^[^ ^169^ ^]^ fabricated SnS_2_ nanoflakes on a PI/PET substrate using atomic layer deposition. The sensor demonstrated high NO_2_ sensitivity across concentrations from 30 to 1 ppm and retained performance after 70,000 bending cycles.^[^ ^169^ ^]^ Furthermore, a liquid metal/SnS_2_ epidermal sensor demonstrated exceptional sensitivity (1092%/ppm) and an ultralow detection limit (1.32 ppb) for NO gas, emphasizing its potential for healthcare applications like respiratory monitoring.^[^ ^170^ ^]^

WOx/WSe_2_ sensors have also shown potential for flexible NO sensing. Medina et al.^[^ ^171^ ^]^ reported their fabrication on PI substrates. While minor sensitivity reductions were observed at bending angles beyond 45°, the sensors‐maintained functionality under significant bending conditions, highlighting their practical applicability.

**Figure 7:**
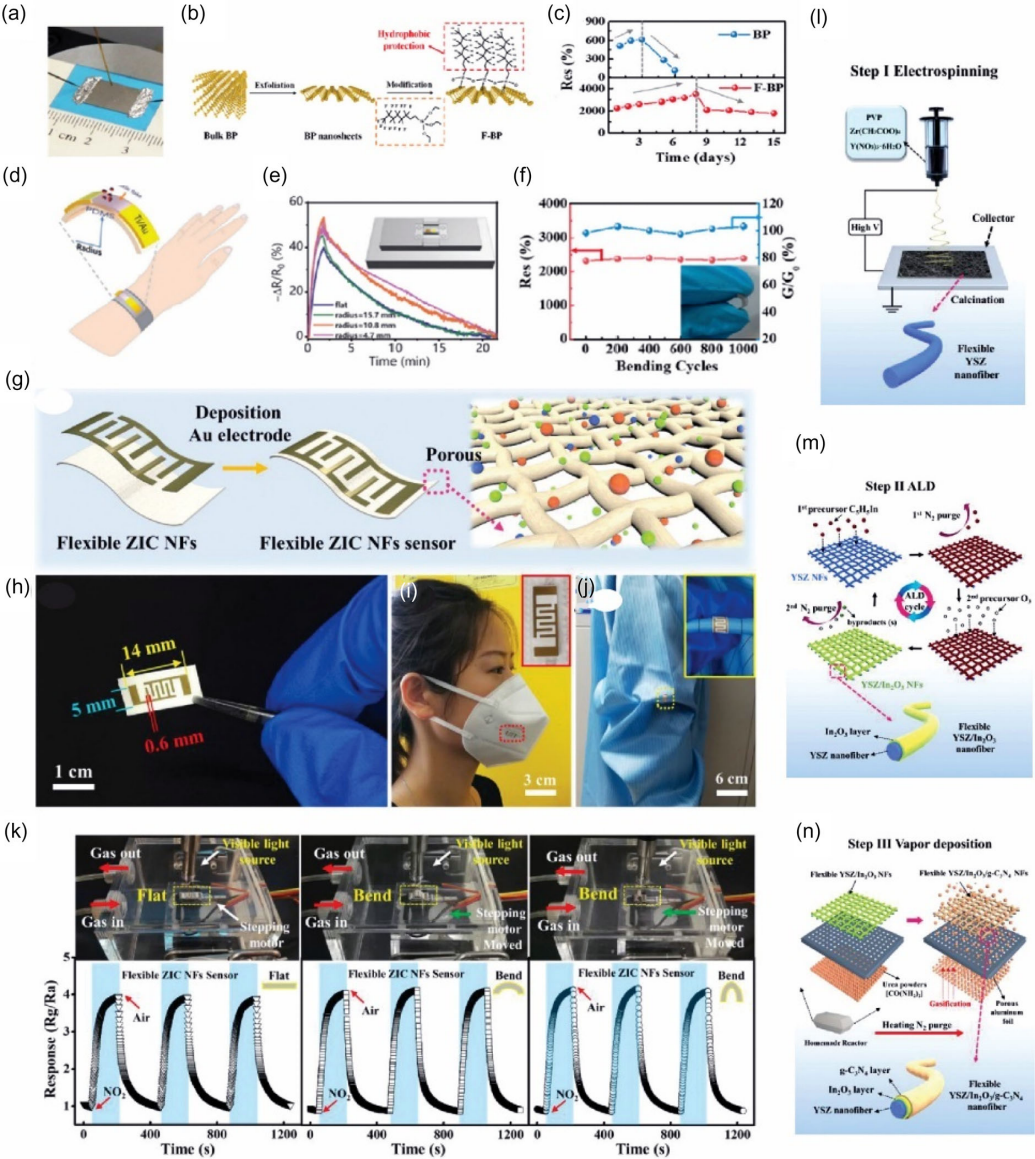
a) Photograph of the flexible BP based gas sensor. Reprinted with permission.^[^ ^3^ ^]^ Copyright 2023, The Royal Society of Chemistry. b) Illustration of BP functionalization using hydrophobic fluoroalkylsilane via salinization. c) Comparison of gas sensing responses for BP and F‐BP‐based sensors. Reproduced with permission.^[^ ^179^ ^]^ Copyright 2021, American Chemical Society d) Diagram of the wearable GaSe‐based NO_2_ sensor design. e) Dynamic response curves showing the relative resistance change over time for a flexible GaSe sensor exposed to 1000 p.p.b. NO_2_ at different curvature radii. Reproduced with permission.^[^ ^172^ ^]^ Copyright 2020, WILEY‐VCH. f) Gas sensing performance and conductivity variations of the flexible sensor after repeated bending cycles. Reproduced with permission.^[^ ^179^ ^]^ Copyright 2021, American Chemical Society. g) Diagram depicting NO_2_ gas permeation and the structure of the flexible ZIC NF sensor. h) Photo of the ZIC NF sensor featuring interdigitated Au electrodes. i) Image showcasing the placement of the ZIC NF sensor on the exterior of a 3M mask. j) Visual of the ZIC NF sensor undergoing bending tests on a clothing surface. k) Dynamic gas‐sensing performance of the flexible ZIC sensor under visible‐light illumination and bending conditions for 200 ppb NO_2_. Diagram of the process for preparing flexible YSZ/In_2_O_3_/g‐C_3_N_4_ NF networks. l) Step I: Electrospinning a precursor solution followed by calcination to produce flexible YSZ NFs. m) Step II: Deposition of In_2_O_3_ layers onto YSZ NFs using the atomic layer deposition (ALD) technique. n) Step III: Vapor deposition of g‐C_3_N_4_ layers on YSZ/In_2_O_3_ NFs to complete the flexible YSZ/In_2_O_3_/g‐C_3_N_4_ NF network. Reproduced with permission.^[^ ^182^ ^]^ Copyright 2020, Wiley‐VCH.

Zhao et al.^[^ ^172^ ^]^ introduced a 2D GaSe‐based sensor for NO_2_ detection, showcasing exceptional sensitivity of 0.5 parts per billion (p.p.b.) at RT, combined with outstanding selectivity ratios exceeding 100 against interfering gases. The sensor maintained stable performance after 10 days of air exposure, highlighting its durability. Practical applications included detecting vehicle exhaust emissions and wearable NO_2_ sensing. To demonstrate GaSe's robust suitability for wearable gas‐sensing applications, a GaSe sensor array was fabricated on a PDMS substrate, as depicted in **Figure** **7**d. A flexibility test was conducted on one of the GaSe sensor units using a customized flexible‐testing platform (Figure 7e, inset). The sensor's performance was evaluated under varying radii of curvature to assess tensile and compressive strain effects derived from bending. Figure 7e  shows the dynamic response curves of relative resistance change versus time for the GaSe sensor exposed to 1000 p.p.b. NO_2_. The sensitivity increased as the bending radius decreased. Specifically, the sensor exhibited a sensitivity of 40% before bending, which improved to 54% when bent to a radius of 4.7 mm. These results underscore the GaSe sensor's flexibility and enhanced performance, making it highly suitable for wearable and practical gas‐sensing applications.

Another interesting development is *Janus* *TMDs* (like MoSSe, which has different atoms on each face) predicted to have even better gas‐sorption properties.^[^ ^173^ ^]^ While not experimentally integrated into flexible devices yet, theoretical studies suggest they could be promising due to built‐in dipoles that attract gas molecules.^[^ ^132^ ^]^

The gas sensing characteristics of 2D stretchable and wearable TMDs‐based gas detectors are summarized in **Table** **3**, and all other 2D materials‐based gas sensor characteristics are summarized in **Table** **4**. These sensors demonstrate the capability to detect a wide range of gases, including NO_2_, NH_3_, CO, and others, showcasing their versatility in various applications. Importantly, the performance of these wearable and flexible gas sensors remains stable under diverse mechanical deformations such as bending at different angles, twisting, and stretching.

**Table 3:** 2D TMDs materials‐based flexible gas sensors.

Sensing material	Substrate	Operating temperature (°C)	Taget gas	Concentration	Response (%)	Response/recovery time	LOD or (range)	Reference
MoS_2_	PI	RT	NO_2_	25 ppm	97	3/8 min	25 ppm	[161]
MoS_2_b	PET	80 °C	NH_3_	400 ppm	33	158/385 s	50 ppm	[232]
MoS_2_	PET	RT	NO_2_	400 ppb	671	16/65 s	20 ppb	[56]
MoS_2_	PDMS	RT	Humidity	90% RH	2600	–	30%–90% RH (range)	[233]
MoS_2_	PDMS	RT	Humidity	35% RH	104	10/60 s	0%–35% RH (range)	[234]
MoS_2_	PET	RT	Humidity	65% RH	2048	–	5%–65% RH (Range)	[227]
MoS_2_	PET	RT	Acetone	120 ppm	16.3	–	–	[235]
MoS_2_‐Au	PET	RT	Acetone	10 ppm	15	≈100/385 s	–	[235]
MoS_2_‐Pt NPs	PET	RT	NO_2_	0.5 ppm	6	>30 min	2 ppb	[236]
Pd‐MoS_2_	PI	150 °C	NH_3_	10 ppm	3	–	–	[237]
MoS_2_‐Cu_2_S	Cellulose paper	27 °C	Humidity	25%−78% RH	40	–	2%	[238]
Graphene/MoS_2_	PI	150 °C	NO_2_	5 ppm	7	–	1.2 ppm	[66]
MoS_2_‐SWCNT	PET	RT	NO_2_	40 ppm	54	–	1.5 ppm	[24]
PANI/MoS_2_	PDMS	RT	NH_3_	30 ppm	75.58	≈150 s	50 ppb	[164]
rGO/MoS_2_	PET	25 °C	Humidity	10% RH	9.03	≈30/253 s	178.3 ppm	[239]
MoS_2_/rGO	PET	90 °C	NO_2_	5 ppm	≈25%	–	0.15 ppm	[163]
rGO/MoS_2_	PEN	RT	HCHO	10 ppm	4.8	–	–	[240]
MoSe_2_	PI	RT	NO_2_	2 ppm	≈20	250/150 s	10 ppb	[56]
MoSe_2_	PI		NH_3_/NO_2_	1 ppm	≈145 (NH_3_), ≈90 (NO_2_)	<200 s	–	[56]
GaSe	PDMS	RT	NO_2_	0.5 ppb	≈40	–	0.5 ppb	[172]
WS_2_	PDMS	RT	Humidity	90% RH	2357	5/6 s	20%−90% RH (range)	[241]
Au‐functionalized WS_2_	PI	60 °C	CO	1 ppm	≈1.2	–	–	[139]
Pd‐WS_2_/Si heterojunction	Ultrathin Si	RT	H_2_	0.1%	≈2	44.7/35.1 s	–	[165]
WS_2_	Filter paper	RT	NH_3_	50 ppm	≈2	–	–	[166]
SnS_2_	PI	RT	NO_2_	1 ppm	≈309	–	–	[169]
SnS_2_/S‐rGO	Cloth, Paper	RT	NO_2_/NH_3_	125 ppb	≈20 (cloth), ≈15 (paper)	–	0.7 ppb (NO_2_) 1.9 ppb (NH_3_)	[138]
SnS_2_	PET, skin	RT	NO	25 ppb	≈22	–	1.32 ppb	[170]
NbSe_2_/WSe_2_	Plastic	–	NO_2_/NH_3_	1 ppm	≈27.5 (NO_2_), ≈2 (NH_3_)	–	–	[168]
WOx/WSe_2_	PI	–	NO_2_	25 ppb	≈20	≈250 s	0.3 ppb	[171]
WSe_2_ nanosheets	PI	RT	TEA/NO_2_	10 ppm	≈5.36 (NO_2_), ≈5 (TEA)	–	8 ppb	[242]

Abbreviations: CO, carbon monoxide; CPI, colorless polyimide; DMMP, dimethyl methylphosphonate; F‐BP, fluorinated black phosphorus; GO, graphene oxide, H_2_, hydrogen; HCHO, formaldehyde; H_3_, ammonia; NO_2_, nitrogen dioxide; PDMS, poly(dimethylsiloxane); PET, polyethylene terephthalate; PI, polyimide; PLLA, poly‐L‐lactic acid; PPB, parts per billion; PPM, parts per million; rGO, reduced graphene oxide; RH, relative humidity; SO_2_, sulfur dioxide; TEA, triethylamine.

**Table 4:** Summary of 2D flexible and wearable gas sensor characteristics.

Sensing material	Substrate	Operating temperature (°C)	Target gas	Concentration	Response (%)	Response/recovery time	LOD or range	Reference
F‐BP	Flexible interdigital electrode	RT	NO_2_	1 ppm	≈2000	42/102 s	–	[179]
In_2_O_3_/g‐C_3_N_4_	YSZ	RT	NO_2_	200 ppb	≈4	–	4–200 ppb (range)	[182]
PANI/g‐C_3_N_4_	PVDF non‐fabric	RT	NO_2_	9 ppm	≈30	–	–	[6]
Au and Pt decorated Ti_3_C_2_Tx	PET	RT	NH_3_	50 ppm	16	–	–	[187]
Ti_3_C_2_Tx	PI	RT	NH_3_	200 ppm	≈0.12	–	–	[243]
Ti_3_C_2_Tx MXene framework	PET	RT	Methanol	5 ppm	≈1.2	2 min	50 ppb	[185]
Ti_3_C_2_/MXene	PET	RT	NH_3_	5 ppm	≈0.55	76.6/17.8 s	–	[108]
Ti_3_C_2_Tx‐F	PI	RT	Ethanol	5.8 ppm	≈0.12	39/139 s	5–120 ppm (range)	[186]
MXene/rGO	Fibrous threads	RT	NH_3_	50 ppm	≈7	–	10–50 ppm (Range)	[189]
V_2_CTx	PI	RT	H_2_	100 ppm	≈0.22	2/7 min	2–100 ppm (range)	[10]
rGO	PET	RT	NO_2_	5 ppm	≈11.5	7/28 min	1–20 ppm (range)	[150]
rGO	Filter paper	RT	NH_3_	400 ppm	≈5 (flat), ≈3.5 (bent)	–	430 ppb	[196]
Ag–S‐rGO	PI	RT	NO_2_/NH_3_	100 ppm	≈90 (NO_2_), ≈3.5 (NH_3_)	12/20 s	0.5–50 ppm (range)	[149]
Ag/rGO	Photo paper	RT	NO_2_	0.2 ppm	≈1.2	120–220 s/‐	0.2–5.0 ppm (range)	[62]
PPy‐rGO composite	Kapton film	RT	NH_3_	10 ppm	≈45	200 s/10 min	41 ppb	[198]
Fe_2_O_3_/rGO	PET	RT	NO_2_	1000 ppb	≈30	14 s/‐	–	[244]
rGO/Pd	PI, paper, textile	RT and 120	H_2_	0.2%	≈4	–	–	[197]
rGO/SnO_2_	PVDF	RT	H_2_	10 ppm	≈49.2	34 s/142 s		[245]
Graphene/MoS_2_	PI	150	NO_2_/NH_3_	20 ppm	≈60 (NO_2_), ≈10 (NH_3_)	–	1.2 ppm NO_2_	[66]
Graphene	PET	RT	NO_2_	200 ppm	≈23 (strain), ≈25 (relax)	–	–	[246]
Graphene	PI	RT	NO_2_	5 ppm	≈13	≈300 s/300 s	1–20 ppm (range)	[20]
Single‐/bi‐layer graphene	PES	RT	NO_2_	**50 ppb**	≈12	14 s/11 s	50 ppb	[202]
LIG	Kevlar/nonwoven fabrics	RT	NO_2_	1 ppm	≈12	40/291 s	6.1 ppb	[204]
CeO_2_‐coated CuBr	PI	RT	NH_3_	5 ppm	≈68	–	–	[21]
rGO/ZnO	Cotton/Elastic Threads	RT	NO_2_	0.5 ppm	≈7.5	140/630 s	43.5 ppb	[77]
In_2_O_3_/Pt	PI	40 and 125	Ethanol	95 ppb	≈25	1/2 s	–	[247]
WO_3_‐δ	PI	RT	NO_2_	10 ppm	≈17	17/25 s	1.8 ppb	[248]
WO_3_‐PEDOT:PSS	PI	RT	NO_2_	50 ppb	≈1.1	45.1/88.7 s	30 ppb	[249]
V_2_O_5_	PLLA	RT	NH_3_	5 ppm	≈1.1	20/16 s		[250]
UiO‐66‐NH_2_	PAN, CNT	30‐70	SO_2_	50 ppm	73.3	255/170 s	1–125 ppm (Range)	[208]
CuBTC/carbon‐graphene composite	PDMS	RT	NH_3_	20 ppm	4.6	–	10–100 ppm (range)	[143]
CuTCA/TiO_2_	PDMS	RT	NO	50 ppm	124	–	140 ppb	[144]
Cu‐BHT	PET	RT	NH_3_	1 ppm	30	58/102‐ s	0.23 ppm	[209]
ZIF‐7 NPs/TiO_2_	PET	RT	Formaldehyde	5 ppm	50	–	0.0038 ppm	[39]
CH_3_NH_3_SnI_3_	cloth	RT	NH_3_	100 ppm	85	–	–	[213]
OIMHP	ITO	RT	NO_2_	1 ppm	6.3	5.7 s/12.7 s,	–	[217]

Abbreviations: Ag/rGO, silver‐decorated reduced graphene oxide; Ag–S‐rGO, silver‐sulfide functionalized reduced graphene oxide; CO, carbon monoxide; CuBTC, copper Benzene‐1,3,5‐tricarboxylate (also known as MOF‐199); CuTCA, copper tricarboxylate; DMMP, dimethyl methylphosphonate; Fe_2_O_3_, Iron (III) Oxide (hematite); g‐C_3_N_4_, graphitic carbon nitride; GO, graphene oxide; H_2_, hydrogen; HCHO, formaldehyde; In_2_O_3_, indium(III) oxide; ITO, indium tin oxide; Methanol, CH_3_OH (implied as VOC target); Mxene, a family of 2D transition metal carbides, nitrides, or carbonitrides; Ti_3_C_2_Tx, surface‐functionalized titanium carbide MXene; NH_3_, ammonia; NO, nitric oxide; NO_2_, nitrogen dioxide; OIMHP, organic–inorganic hybrid material (context dependent); PAN, polyacrylonitrile; PANI, polyaniline; PDMS, poly(dimethylsiloxane); PEDOT,PSS, Poly(3,4‐ethylenedioxythiophene),poly(styrenesulfonate); PEN, polyethylene naphthalate; PES, polyethersulfone; PET, polyethylene terephthalate; PI, polyimide; PLLA, poly‐L‐lactic acid; PPB, parts per billion; PPM, parts per million; Ppy, Polypyrrole; PVDF, polyvinylidene fluoride; rGO, reduced graphene oxide; RH, relative humidity; RT, room temperature; SO_2_, sulfur dioxide; TEA, triethylamine; Ti_3_C_2_Tx‐F, fluorinated titanium carbide MXene; UiO‐66‐NH_2_, amino‐functionalized metal‐organic framework (MOF) UiO‐66; V_2_CTx, surface‐functionalized vanadium carbide MXene; V_2_O_5_, vanadium pentoxide; WO_3_, tungsten trioxide; WO_3_‐δ, oxygen‐deficient tungsten trioxide; ZIF‐7, zeolitic imidazolate framework‐7.

The advantages are as follows: high surface area with abundant active sites, intrinsic semiconducting properties enabling room‐temperature operation, and excellent mechanical flexibility (maintaining performance even after bending),^[^ ^3^, ^174^ ^]^ well‐suited for low‐power, on‐body environmental monitors due to strong responses at ambient conditions.

The limitations are as follows: slow baseline recovery and hysteresis unless assisted by UV light or heating (e.g., MoS_2_ needed UV desorption).^[^ ^175^ ^]^ Environmental stability can be an issue for some TMDs—while MoS_2_ and WS_2_ are stable, others like black arsenic–phosphorus oxidize faster. Achieving uniform large‐area films on flexible substrates can be challenging (although printing and solution‐coating are mitigating this). Repeatability and sensor‐to‐sensor variation, as well as selectivity without functionalization, remain concerns for TMDs based sensors.^[^ ^3^ ^]^

### 3.2 BP‐Based Flexible Gas Sensors

BP (phosphorene in monolayer form) has drawn attention for gas sensing because of its direct bandgap (≈0.3–0.35 eV) and high carrier mobility, which together yield exceptionally large sensor responses. BP is a *p‐type* semiconductor; upon exposure to oxidizing gases like NO_2_, BP readily accepts electrons (holes increase), leading to pronounced current changes.^[^ ^176^ ^]^ Indeed, BP FET sensors have achieved NO_2_ detection down to tens of parts‐per‐trillion in controlled conditions (e.g., ≈0.2 ppb reported) and a response over 100% at 5 ppb NO_2_—outperforming many other 2D materials in baseline sensitivity. BP's layered structure is also mechanically flexible, so BP thin‐film sensors can be incorporated onto flexible substrates. However, the *Achilles’ heel* of BP is its rapid degradation in ambient atmosphere (forming P_x_O_y_ and losing conductivity).^[^ ^176^ ^]^ This instability historically limited BP's practical use, but recent high‐impact work shows that chemical modification can “stabilize” BP for real‐world sensing.

As a 2D material, BP offers a high surface area, high carrier mobility, low out‐of‐plane conductivity, and high surface reactivity, making it a promising candidate for gas sensing applications (Figure 7a). However, its chemical stability in normal atmospheric conditions remains a significant challenge.^[^ ^177^ ^]^ BP has demonstrated excellent selectivity for paramagnetic gas molecules such as NO_2_, making it valuable for gas sensors.^[^ ^136^ ^]^ Theoretical calculations by Kou et al.^[^ ^178^ ^]^ further confirmed BP's ability to adsorb various gases, including NO, NO_2_, CO, CO_2_, and NH_3_.

Building upon advancements in enhancing material stability for flexible sensors, Xu et al.^[^ ^179^ ^]^ achieved *simultaneous improvement of* *BP* *chemical stability and* *NO* *_2_ sensitivity* by attaching fluoroalkylsilane molecules to BP's surface (essentially sealing reactive edge sites with ^−^F terminated silane) (Figure 7b). The silane‐treated BP became hydrophobic (preventing water‐induced oxidation) and retained performance under 5%–95% relative humidity. In fact, the silanized BP's response to 100 ppb NO_2_ was ≈3.9× higher than untreated BP, and its response remained constant across a broad humidity range, a crucial improvement for wearable use. Furthermore, the sensor retained its stable response to 1 ppm NO_2_ even after 1000 bending cycles at a bending angle of 45° (Figure 7f), demonstrating its robustness and durability under mechanical stress. Another strategy is metal decoration: Valt et al.^[^ ^176^ ^]^ reported an air‐stable BP sensor by decorating BP flakes with Ni NPs. The Ni acts as an oxygen scavenger and forms a partial charge‐transfer junction with BP. The resulting Ni–BP sensor showed ≈100% sensing response even at 0.1 ppm NO_2_ in air, with negligible performance degradation over 4 weeks in ambient conditions. This is a remarkable improvement given that pristine BP would degrade within hours to days without protection. Similar approaches using noble metals or metal oxides (e.g., Pt, Au, or α‐MoO_3_ on BP) have also yielded composite sensors that marry BP's sensitivity with a degree of environmental robustness.

With these advancements, BP‐based sensors can be considered for wearable and flexible devices. For instance, a BP sensor film encapsulated by an ionogel or parylene could serve as a flexible breathing monitor for detecting NO_2_ or NH_3_ in exhaled breath—taking advantage of BP's high sensitivity to amine and oxidizing gases. One demonstration involved BP integrated on a PI substrate with an epoxy coating: It remained operational for days in ambient air, detecting NO_2_ reliably at the ppm level.^[^ ^179^ ^]^ BP's anisotropic structure might also enable directional sensing (responding differently to strain in different directions), which is a unique property among 2D materials.

The advantages are as follows: exceptional intrinsic sensitivity (able to detect sub‐ppb gas concentrations) and fast response due to high mobility.^[^ ^176^ ^]^ BP offers a different selectivity profile (strong affinity for NO_2_, H_2_S, etc.) that complements n‐type materials. Flexible BP devices are feasible since BP can be exfoliated in nanosheets and transferred to polymers.

The limitations are as follows: Very poor ambient stability without protection, BP must be passivated (via functionalization encapsulation, or composite design) for any practical use. Even with stabilization, long‐term durability under UV exposure or high humidity is still being studied. Another limitation is potential toxicity of phosphorus oxides formed upon BP degradation (though in small sensors this is minor). BP flakes also tend to vary in thickness and size from preparation, which can introduce device variability.

### 3.3 2D Nitride Nanocomposite Materials‐Based Flexible Gas Sensor

This category primarily involves *graphitic carbon nitride (g‐C_3_N_4_)* and related 2D nitrides, often used as nanocomposites for gas sensing. Pure g‐C_3_N_4_ is a metal‐free 2D network of C and N with a moderate bandgap (≈2.7 eV) and is inherently *insulating to semiconducting*, which means that it usually requires modification to be used as a chemiresistive sensor.^[^ ^180^, ^181^ ^]^ On the upside, g‐C_3_N_4_ is chemically robust (resistant to oxidation up to ≈500 °C), environmentally benign, and can be produced cheaply in bulk (by thermal polymerization of precursors like urea). These features make it attractive for flexible sensors in principle—especially as a *matrix or additive* that can impart selectivity and stability. Recent research has focused on combining g‐C_3_N_4_ with conductive materials to create hybrid sensing layers. Such composites harness g‐C_3_N_4_'s high density of surface functional groups (amine and triazine nitrogen sites) that can interact with gas molecules, while the added conductor provides a percolation network for electrical readout.^[^ ^180^, ^181^ ^]^ A prime example is a *g‐C_3_N_4_/metal oxide composite*: Han et al.^[^ ^182^ ^]^ developed a highly flexible, all‐inorganic gas sensor using an ultrathin heterostructure of In_2_O_3_/g‐C_3_N_4_ as the active sensing layer and yttria‐stabilized zirconia (YSZ) nanofibers (NFs) as the substrate. The preparation process of flexible YSZ/In_2_O_3_/g‐C_3_N_4_ NF networks is shown in Figure 7l–n. This configuration resulted in a flexible YSZ/In_2_O_3_/g‐C_3_N_4_ (ZIC) gas sensor, designed to address the demands of wearable environmental sensing and respiratory health diagnostics. The sensor's viability was tested by directly depositing gold (Au) interdigitated electrodes onto the as‐prepared ZIC network, as illustrated in Figure 7h. The fibrous ZIC network also provided high permeability, a critical advantage for wearable applications. To further demonstrate its practicality, the ZIC sensor was integrated into a face mask and experimental garments, as shown in Figure 7i,j, successfully conforming to the deformation of these substrates. The sensor exhibited outstanding adaptability, maintaining reliable performance under various bending conditions and visible‐light exposure. It demonstrated a strong response to NO_2_ concentrations ranging from 4 to 200 ppb (Figure 7k), emphasizing its potential for real‐world applications in wearable respiratory diagnostics and environmental monitoring. This work underscores the promise of flexible, freestanding gas sensors as innovative solutions for portable and wearable sensing technologies.

Another example includes a highly flexible and wearable NO_2_ sensor developed by Khalifa and Anandhan,^[^ ^6^ ^]^ employing an electrospun poly(vinylidene fluoride) (PVDF)/PANI/g‐C_3_N_4_ nanocomposite. This sensor exhibited a 47% response to 25 ppm of NO_2_ at RT. The sensor retained its sensitivity after 50 bending cycles, with minimal response degradation during bending tests. The high tensile strength and flexibility of the electrospun NFs contributed to its durability and consistent performance.

The role of g‐C_3_N_4_ in these composites can also be to target specific gases. Its surface amine groups have affinity for acidic gases like NO_2_ and H_2_S via acid–base interactions, and its π‐conjugated domains can interact with aromatic VOCs. On its own, g‐C_3_N_4_ has been used in optical gas sensing (e.g., PL quenching by oxygen). But for flexible electronics, the conductive composite approach is more relevant. These composites can be processed in solution (g‐C_3_N_4_ is often obtained as few‐layer nanosheets via exfoliation), allowing techniques like drop‐casting or screen‐printing onto flexible substrates. For instance, researchers have printed g‐C_3_N_4_/WO_3_ ink on paper to create a disposable ammonia sensor, and the addition of g‐C_3_N_4_ was found to lower the optimum operating temperature of WO_3_ (toward RT) by enhancing NH_3_ adsorption at the surface.^[^ ^183^ ^]^

The advantages are as follows: excellent chemical and thermal stability, with low toxicity—ideal as a *support material* in sensors. In thin‐film form, g‐C_3_N_4_ is mechanically flexible and can be integrated onto textiles or plastics without losing functionality. It introduces selectivity via its functional groups (e.g., strong adsorption of electron‐seeking gases) and can improve sensor linearity and humidity tolerance when used in composites. Also, g‐C_3_N_4_ is inexpensive and amenable to large‐scale fabrication (even printing),^[^ ^183^ ^]^ aligning with the needs of wearable devices.

The limitations are as follows: Poor electrical conductivity of pure g‐C_3_N_4_ means that it usually cannot serve as the sole sensing channel—a percolating conductive network (like carbon nanotubes, graphene, or a doped polymer) is needed to transduce the signal. This requirement adds complexity to sensor fabrication. Additionally, g‐C_3_N_4_ alone has relatively slow response/recovery kinetics at RT because gas adsorption on its surface is mostly physisorptive; it may require UV light or mild heating to accelerate reactions (many g‐C_3_N_4_ sensors are studied under UV illumination due to its photocatalytic nature). Finally, while g‐C_3_N_4_ is generally stable, in composite form, the overall stability will depend on the other component (e.g., a g‐C_3_N_4_/metal oxide sensor could inherit the moisture sensitivity of the oxide). Thus, careful design is needed to ensure long‐term reliability.

### 3.4 2D MXenes‐Based Flexible Gas Sensor

MXenes, a novel class of 2D materials, possess exceptional electrical properties, high surface areas, tunable bandgaps, and abundant adsorption sites, making them ideal candidates for flexible gas sensors. Their general formula, M_n+1_X_n_T_x_, where M is a transition metal, X represents carbon or nitrogen, and T_x_ denotes surface functional groups (e.g., ^−^O, ^−^OH, ^−^F, ^−^Cl), highlights their unique structure and chemical versatility.^[^ ^184^ ^]^ The first member of this family, Ti_3_C_2_T_x_, was synthesized by etching aluminum from Ti_3_AlC_2_, paving the way for extensive exploration of MXene‐based materials.^[^ ^37^, ^185^ ^]^

Leveraging these unique properties, MXene‐based gas sensors are known for their sensitivity to VOCs at RT. For example, a Ti_3_C_2_T_x_ sensor on a PI substrate demonstrated high ethanol sensitivity within the 6–30 ppm range, maintaining stable performance even after 1000 bending cycles.^[^ ^186^ ^]^ Building on this, functionalization plays a key role in enhancing their gas‐sensing properties. Li et al.^[^ ^137^ ^]^ reported significant improvements in ethanol sensitivity by replacing ^−^F terminations on Ti_3_C_2_T_x_ with trimethylacetic anhydride resulted in a fivefold increase in ethanol sensitivity and a 71% reduction in humidity interference, emphasizing the critical role of surface chemistry.

Beyond intrinsic and chemically modified MXenes, the catalytic properties of noble metals, such as Au and Pt, further enhance MXene performance. Ti_3_C_2_T_x_ sensors decorated with these metals exhibited excellent ammonia (NH_3_) selectivity, leveraging Schottky junction formation and catalytic effects. These sensors maintained their functionality after extensive mechanical deformation, including 5000 bending and 1000 tilting cycles, and operated efficiently at low voltages.^[^ ^187^ ^]^

**Figure 8:**
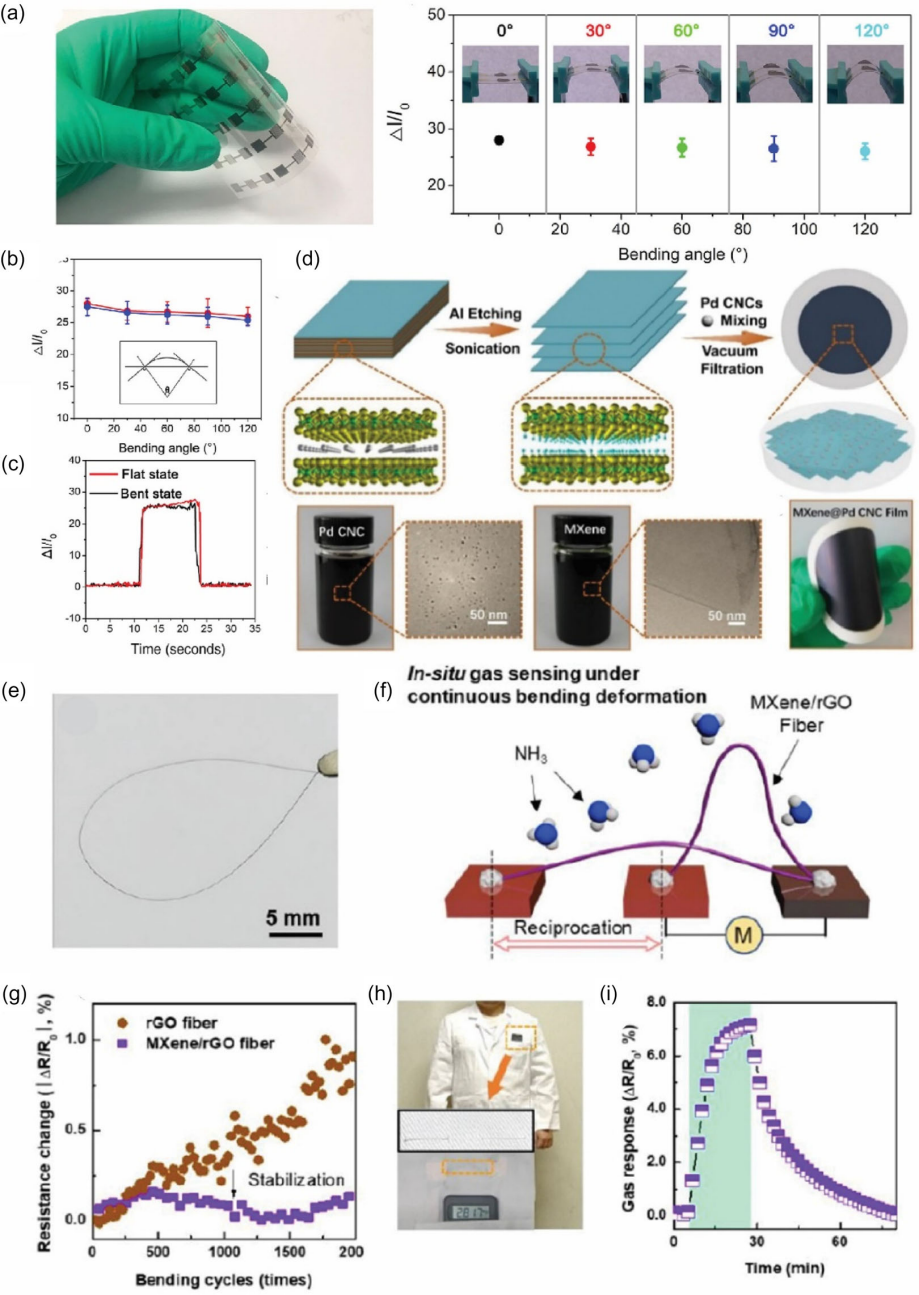
a) Optical images of flexible gas sensors fabricated using PANI/Ti_3_C_2_Tx. b) Sensitivity of PANI/Ti_3_C_2_Tx‐based sensors towards 150 ppm ethanol as a function of bending angle (*n* = 3 experiments). c) Transient sensing response of PANI/Ti_3_C_2_Tx ethanol gas sensors under various bending angles (0° to 120°) at 150 ppm concentration. Reproduced with permission.^[^ ^188^ ^]^ Copyright 2019, Wiley‐VCH. d) Schematic illustration of the synthesis process for an MXene@Pd CNC film, Photographic images of Pd CNC and MXene suspensions, including inset optical micrographs on glass slides, Photographic view of the assembled sensor. Reproduced with permission.^[^ ^141^ ^]^ Copyright 2020, Elsevier. e) Image demonstrating the bendability and flexibility of MXene/rGO hybrid fibers (40 wt% MXene). f) Schematic representation of fiber bending measurements. g) Comparative analysis of resistance to cyclic bending fatigue for pristine rGO fibers versus MXene/rGO hybrid fibers. h) Lab coat integrated with MXene/rGO hybrid fibers, shown with a multimeter to highlight conductivity. i) Gas sensing response of MXene/rGO hybrid fibers woven into a lab coat to 100 ppm NH_3_ gas molecules. Reproduced with permission.^[^ ^189^ ^]^ Copyright 2020, American Chemical Society.

Extending the material design strategies, Zhu et al.^[^ ^141^ ^]^ developed a flexible, lightweight hydrogen (H_2_) sensor using Ti_3_C_2_Tx MXene nanosheets decorated with palladium (Pd) colloidal nanoclusters (CNCs). Fabricated via a simple vacuum‐filtration process, the MXene@Pd CNC paper‐based sensor demonstrated excellent adaptability and a compact, glossy design (**Figure** **8**d). The sensor showed reliable H_2_ detection at RT in both flat and bent states, with a response time of (32 ± 7) s and a sensitivity of (23.0 ± 4.0)% at 4% H_2_. Furthermore, it supported “in‐situ‐mode” detection directly on the paper film, enabling real‐time monitoring. The enhanced performance resulted from strong H_2_ adsorption by Pd CNCs, which altered the work function and induced electron doping in the MXene. This efficient and versatile design highlights the potential of MXene@Pd CNC sensors for next‐generation portable and wearable gas‐sensing technologies. While this work demonstrated the integration of MXenes with noble metal nanoclusters to enhance H_2_ sensing, other strategies focus on incorporating conductive polymers to further improve mechanical compliance and gas permeability for wearable settings.

To address the limitations of inorganic‐only systems, Zhao et al.^[^ ^188^ ^]^ engineered a hybrid Ti_3_C_2_Tx/PANI sensor that not only enhanced ethanol sensitivity but also sustained excellent flexibility under mechanical bending, showing the synergistic benefits of Mxene‐polymer interfaces. The Ti_3_C_2_Tx/ PANI composites based sensor achieved a sensitivity of 27.4% to 150 ppm ethanol with rapid response and recovery times of 0.6 and 0.8 s, respectively. These sensors maintained excellent performance under mechanical bending up to 120°, showcasing robust flexibility and durability. Figure 8a left side depicts the flexible sensor design, with its adaptability evident through stable relative current changes across bending angles (Figure 8a, right). The sensor consistently retained high sensitivity, outperforming pristine Ti_3_C_2_Tx sensors, which showed a slight performance decline under similar conditions. Figure 8b highlights the sensor's stable response across bending angles, and after 140 bending cycles at 60°, it demonstrated negligible changes in response and recovery rates (Figure 8c). This underscores the sensor's mechanical resilience and suitability for dynamic wearable applications.

Furthermore, structural hybridization of MXenes with other 2D materials has been pursued to improve mechanical robustness and sensing performance. Lee et al.^[^ ^189^ ^]^ developed hybrid fibers of Ti_3_C_2_Tx and rGO using a wet‐spinning method, demonstrating stable NH_3_ detection even after 2000 bending cycles. These fibers were seamlessly integrated into wearable devices, such as lab coats, without any performance compromise. Figure 8e presents a photograph highlighting the flexibility and bendability of an MXene/rGO fiber (40 wt% MXene). The experimental setup for evaluating structural stability under repeated bending deformations is illustrated schematically in Figure 8f. The results (Figure 8g) reveal minimal resistance variation for MXene/rGO hybrid fibers during 2000 bending cycles, while rGO fibers exhibited significant resistance fluctuations. This suggests that MXene/rGO hybrid fibers are mechanically and electrically stable, even under bending radii varying from 1 to 0.5 cm. To evaluate wearability, MXene/rGO hybrid fibers were woven into a lab coat and connected to a multimeter, as shown in Figure 8h. When exposed to 100 ppm of NH_3_ gas, the hybrid fibers demonstrated a robust gas response of 7.21% (Figure 8i), confirming their reliability as sensing materials. The excellent mechanical stability, sensitivity to gas molecules, and integration capability of MXene/rGO hybrid fibers underscore their potential as a platform for next‐generation wearable and portable gas‐sensing applications. This aligns with the earlier findings on the adaptability and performance of MXene‐based sensors in wearable contexts.

Complementing VOC and hydrogen sensing applications, MXene‐based sensors have also shown promise for hazardous gas detection. Quan et al.^[^ ^73^ ^]^ developed a paper‐based Ti_3_C_2_T_x_/WS_2_ composite sensor with a response of 15.2% to 1 ppm NO_2_, significantly outperforming standalone components. Other MXenes, such as V_2_CT_x_ and V_4_C_3_T_x_, have shown promising results. V_2_CT_x_ demonstrated effective H_2_ detection on a PI substrate,^[^ ^10^ ^]^ while V_4_C_3_T_x_ sensors responded to 100 ppm acetone with sensitivities of 2.5 at RT and 3.1 at 100 °C.^[^ ^190^ ^]^ Functionalization with 3‐aminopropyl triethoxysilane (APTES) further improved Nb_2_CT_x_ sensitivity and stability for NO_2_ detection at RT.^[^ ^191^ ^]^

These studies collectively demonstrate that the intrinsic 2D nature, tunable surface chemistry, and mechanical flexibility of MXenes make them exceptionally suitable for flexible gas sensor applications.

The advantages are as follows: Metallic conductivity yields low intrinsic noise and large output signals (e.g., tens of percent resistance change).^[^ ^192^ ^]^ MXenes operate at RT and respond quickly, thanks to rapid charge transfer at their surfaces. They are inherently flexible—Ti_3_C_2_T_x_ can be coated as thin films or inks on papers, polymers, and fabrics without cracking. MXenes also offer surface tunability; by controlling termination groups or interlayer spacing (via intercalation), one can modulate their sensitivity and selectivity. Importantly, many MXene sensors can be integrated into wearable platforms (like threads or printable circuits) due to solution processability.

The limitations are as follows: *oxidation and humidity sensitivity*—Ti_3_C_2_T_x_ gradually oxidizes to TiO_2_ in ambient humidity, which can drift sensor baselines over time. Strategies like storing in anhydrous conditions or adding antioxidants help, but long‐term stability is still under study. Moreover, MXenes’ hydrophilicity means that humidity can cause large background resistance changes, requiring either calibration or humidity filters in practical devices. Selectivity can be an issue as well: MXenes tend to interact with any polar molecules (including water vapor), so distinguishing target gases often necessitates either functionalization (e.g., enzyme coatings for biosensing specific analytes) or use in sensor arrays. Finally, while MXene inks are printable, uniform large‐scale fabrication might face challenges such as flake restacking and alignment, which researchers are addressing by printing techniques and additives.

### 3.5 Graphene‐Based Materials (GO, rGO, and Graphene) for Flexible Gas Sensors

GO, rGO, and pristine graphene are pivotal 2D carbon materials explored for flexible and wearable gas sensor technologies. While structurally related, these materials exhibit distinct properties that significantly influence their practical applicability, scalability, and performance under mechanical deformation.

GO, produced by chemical oxidation of graphite, offers the advantages of scalable, low‐cost production and strong hydrophilicity due to abundant oxygen functional groups.^[^ ^145^ ^]^ These functional groups, however, severely limit electrical conductivity, posing a major obstacle for flexible gas sensing applications at RT.^[^ ^68^ ^]^ Although some humidity sensors based on GO have been demonstrated,^[^ ^193^, ^194^ ^]^ there are currently no widely adopted flexible gas sensors employing pristine GO due to its high resistance and poor electron transport at RT.

To overcome GO's limitations, rGO has been developed by partial removal of oxygen groups via chemical or thermal reduction.^[^ ^125^, ^195^ ^]^ rGO retains a defective yet conductive sp^2^ carbon framework, offering markedly improved electrical properties while maintaining flexibility. Several studies have demonstrated the use of rGO for flexible gas sensing. Su and Shieh^[^ ^150^ ^]^ reported a covalently anchored rGO NO_2_ sensor on PET substrates that maintained performance under mild bending but exhibited a 48% drop in response at a 50° angle. Ghosh et al.^[^ ^196^ ^]^ developed an rGO‐based NH_3_ sensor on filter paper, which maintained stable performance about 3% and 13% responses to 400 and 1200 ppm NH_3_, respectively, even under both flat and mechanical deformation, highlighting the mechanical adaptability of rGO networks.

**Figure 9:**
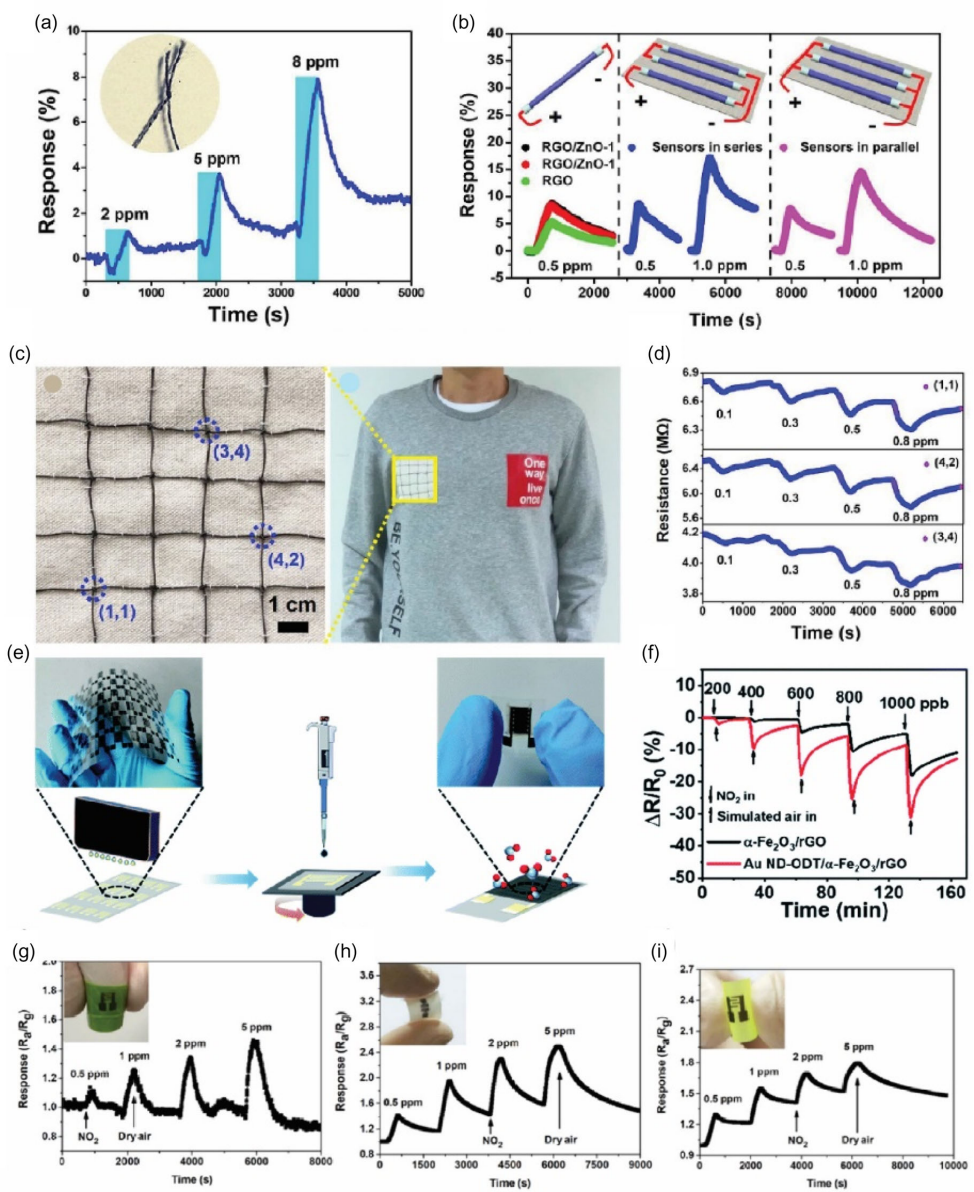
a) Gas‐sensing characteristics of an integrated sensor formed by connecting fragmented CT sensors in series and parallel configurations, highlighting enhanced gas‐sensing performance. b) Gas‐sensing features of individual CT sensors and the combined effects of serial and parallel integration. Images depict the scalable application of woven networks. c) Networks of 4 × 4 ET sensor arrays weaved into a fabric, with a photograph showing sensors stitched into dressings for wearable applications. d) Gas‐sensing characteristics of different sensor modules within the 4 × 4 ET array. Reproduced with permission.^[^ ^77^ ^]^ Copyright 2019, American chemical society. e) Schematic depiction of the fabrication of a flexible NO_2_ sensor with images of printed Ag electrode arrays and the completed gas sensor coated with Au ND–ODT/α‐Fe_2_O_3_/rGO. f) Dynamic response–recovery curves of α‐Fe_2_O_3_/rGO and Au ND–ODT/α‐Fe_2_O_3_/rGO for various NO_2_ concentrations at RT. Reproduced with permission.^[^ ^200^ ^]^ Copyright 2022, The royal society of chemistry. g–i) Responses of flexible NO_2_ gas sensors fabricated on diverse substrates, including (g) a leaf, (h) silk, and (i) a portable sticker, with concentration ranges from 0.5–5 ppm of NO_2_. Insets show images of gas sensors integrated onto a living plant leaf, stitched into a lab coat, and affixed as a wearable sticker. Reproduced with permission.^[^ ^62^ ^]^ Copyright 2018, American chemical society.

Metal NP decoration further enhances rGO's sensing capabilities. Huang et al.^[^ ^149^ ^]^ fabricated an Ag–S–rGO‐based NO_2_ sensor on a PI substrate, achieving 74.6% sensitivity with a rapid 12/20 s response/recovery time under 50 ppm NO_2_ at RT. The sensor demonstrated only a 2.5% resistance increase after 1000 bending cycles at a 1 cm radius. Similarly, Li et al.^[^ ^62^ ^]^ designed an Ag‐decorated rGO NO_2_ sensor integrated onto flexible platforms such as silk fabric, plant leaves, and photo paper. Among these, the silk‐based sensor demonstrated the highest response, while the leaf‐based sensor exhibited the fastest recovery, aided by moderate ambient humidity (**Figure** **9**g–i). The device showed negligible performance degradation even after 3000 bending cycles, confirming its durability for wearable applications.

rGO's versatility has also enabled its use on textile‐based sensors. Further improving durability, Zhu et al.^[^ ^197^ ^]^ fabricated Pd‐decorated rGO hydrogen sensors on textiles, achieving six times greater response than on PI, attributed to the increased surface area and porosity of textile substrates. These sensors maintained stable sensing capabilities after 10,000 bending cycles, demonstrating their durability.

Composite strategies have further improved rGO‐based sensor performance. Gao et al.^[^ ^198^ ^]^ developed a PPy/rGO composite NH_3_ sensor achieving 45% response to 10 ppm NH_3_ at RT, benefiting from efficient electron transfer at polymer‐rGO interfaces. However, challenges remain with metal oxide (MO) composites: For example, Li et al.^[^ ^199^ ^]^ reported an rGO/In_2_O_3_ NO_2_ sensor where weak adhesion between the composite and PI substrate led to compromised flexibility. In contrast, Wang et al.^[^ ^200^ ^]^ successfully fabricated an Fe_2_O_3_‐coated rGO NO_2_ sensor on PET, demonstrating stable sensing performance even after bending angles of 15° and 30°, although higher bending angles (45° and 60°) led to slight degradation (Figure 9e–f). Among the most robust designs, Li et al.^[^ ^77^ ^]^ introduced a highly durable rGO/mesoporous ZnO nanosheet (NS) sensor for NO_2_ detection at RT. Mounted on cotton and elastic thread substrates, the sensor withstood up to 3000 bending cycles, 1000 twisting cycles, and 65% tensile strain without performance degradation. As shown in Figure 9a–d, fiber fractures, a typical failure mode for wearable devices, were mitigated by simple mechanical stitching, enabling repairability. Furthermore, integrating multiple cotton thread (CT) sensors into a fabric matrix significantly enhanced stability and gas‐sensing reliability, paving the way for real‐world wearable sensing applications.

Critically assessing these results, while rGO offers excellent mechanical flexibility, moderate conductivity, and high adaptability to scalable manufacturing (e.g., printing, weaving into fabrics), its sensing performance especially sensitivity and selectivity can lag behind pristine graphene unless enhanced via functionalization.

**Figure 10:**
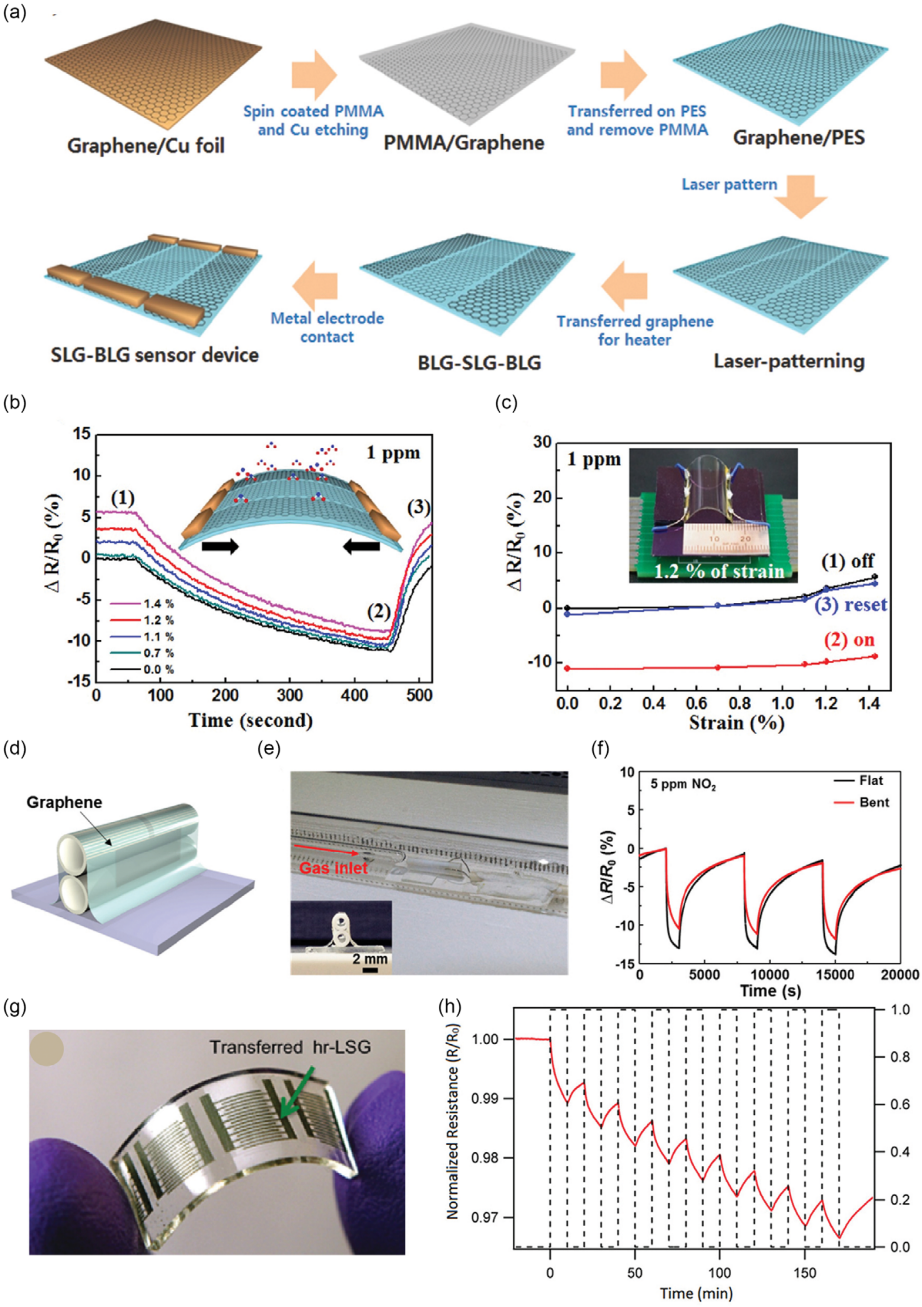
a) Graphene gas sensor and integrated heater fabricated on a transparent and flexible film. b) Dynamic resistance response of single‐layer graphene (SLG) channels under exposure to 1 ppm NO_2_, coupled with a recovery step from 100 to 165 °C and bending strain ranging from 0–1.4%. The inset displays the strained graphene sensor. c) The sensor's operation phases under 1 ppm NO_2_ gas exposure included: 1) off‐state, 2) on‐state, and 3) reset under initial, saturated, and fully recovered conditions, respectively. The inset also illustrates a graphene sensor‐heater junction bent under 1.2% strain. Reproduced with permission.^[^ ^202^ ^]^ Copyright 2014, Wiley‐VCH. d) Schematic representation of an al‐graphene sensor, patterned and coupled to ballpoint pen leads. e) Graphene gas sensor fabricated on a PI substrate. f) Sensor response curves under bending and flat conditions, demonstrating stability in varying mechanical configurations. Reproduced with permission.^[^ ^20^ ^]^ Copyright 2015, Wiley‐VCH). g) Transfer of the same interdigitated electrodes onto PDMS for enhanced flexibility. h) NO_2_ detection using flexible, all‐organic interdigitated electrodes composed of hr‐LSG as active materials. The active electrodes operate in a slightly reduced graphite oxide medium, with NO_2_ detection tested at 20 ppm in dry air. Reproduced with permission.^[^ ^14^ ^]^ Copyright 2012, American chemical society.

Comparatively, pristine graphene exhibits superior intrinsic properties: extremely high carrier mobility (≈2 × 10^5^ cm^2^ V^−1^ s at RT), Young's modulus (≈1 TPa), thermal conductivity (≈5000 W m^−1^·K), and transparency (≈97.7%).^[^ ^7^, ^201^ ^]^ These attributes underpin graphene's extraordinary mechanical resilience and sensing capabilities. Choi et al.^[^ ^202^ ^]^ developed a transparent NO_2_ sensor based on laser‐patterned single‐ and bilayer graphene on PES, achieving 26 s response time to 1 ppm NO_2_ and retaining performance under 1.4% strain and repeated bending cycles (**Figure** **10**a–c).

Self‐heated flexible graphene gas sensors fabricated on PI, as shown by Kim et al.^[^ ^20^ ^]^ offered fast response to 1–10 ppm NO_2_ and endured tight bending radii (1 mm) with only a 3% decrease in response (Figure 10d–f). LIG fabrication offers additional benefits by enabling direct graphene patterning on polymer substrates without transfer steps, improving mechanical integration. Vivaldi et al.^[^ ^203^ ^]^ demonstrated the potential of LIG with functionalized architectures for wearable electronics, while Strong et al.^[^ ^14^ ^]^ developed highly reduced LSG flexible sensors, although initial gas response still required optimization (Figure 10g,h).

Flexible graphene sensors decorated with Ag NPs maintained NO_2_ sensing performance after 1500 bending cycles when integrated into textiles, indicating suitability for breathable wearable applications.^[^ ^204^ ^]^ Printed graphene‐based sensors have also shown promise, although challenges such as poor film uniformity and low pattern resolution persist.^[^ ^203^, ^204^ ^]^ Dopamine‐functionalized graphene e‐textiles exhibited outstanding durability, surviving multiple washing cycles without performance degradation.^[^ ^205^ ^]^

Importantly, while pristine graphene sensors offer the highest sensitivity and mechanical robustness, scalable and cost‐effective production remains a bottleneck. Transferring high‐quality graphene onto flexible substrates without inducing defects or wrinkles remains a technical challenge. On the other hand, rGO's ease of processing, printability, and adaptability to composite architectures provides strong advantages for commercial wearable sensor production, despite its relatively lower sensitivity compared to pristine graphene.

Real‐world deployment of these material systems demands addressing durability under mechanical cycling, humidity robustness, biocompatibility, and seamless integration with energy sources and data transmission units.

In summary, while GO remains primarily a precursor material due to its poor conductivity, both rGO and pristine graphene offer complementary pathways for the realization of high‐performance flexible gas sensors. rGO composites, particularly with conducting polymers or MO nanostructures, present scalable, durable, and cost‐effective options, whereas pristine graphene leads in ultimate sensing performance but at the cost of fabrication complexity. Future advances will depend on overcoming current limitations through hybrid material design, substrate engineering, and system‐level integration, thereby bridging laboratory demonstrations with real‐world, wearable environmental monitoring and health applications.^[^ ^203^, ^204^ ^]^

The advantages are as follows: Graphene‐based materials provide substantial advantages, including high electrical conductivity and sensitivity, excellent mechanical durability, easy integration with diverse flexible substrates, and scalable production methods, particularly for rGO and composites.

The limitations are as follows: However, they also present limitations, such as performance drift over prolonged exposure, challenges in scalable high‐quality graphene production, sensitivity to environmental interference (e.g., humidity), and the need for additional strategies such as functionalization, encapsulation, or composite designs to enhance selectivity and long‐term stability. Continued advancements in composite materials, scalable fabrication techniques, and effective encapsulation will be crucial to fully exploit the potential of graphene‐based materials in widespread wearable and flexible gas‐sensing technologies.

### 3.6 2D MOFs

**Figure 11:**
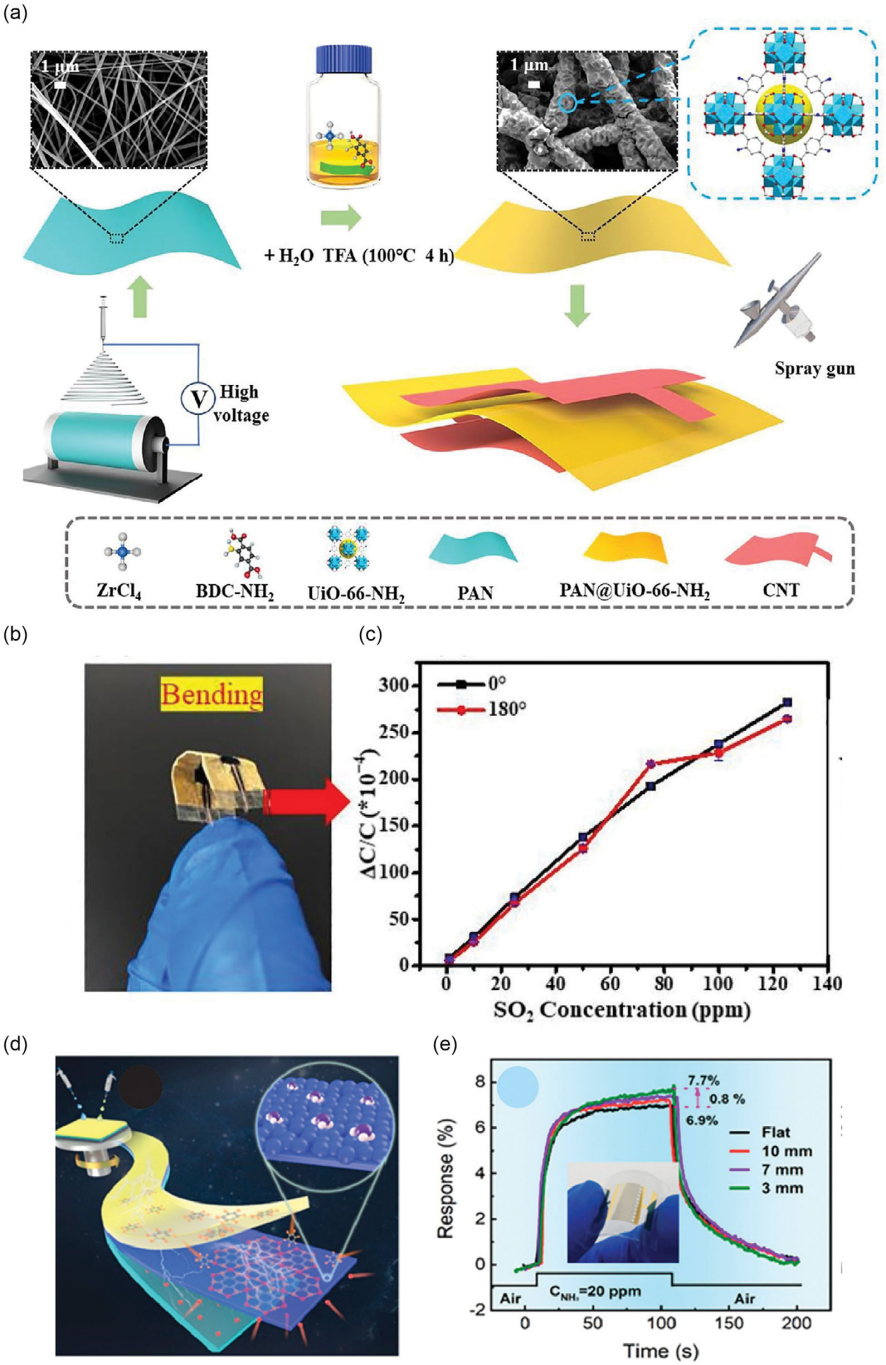
a) Schematic representation of the preparation process for the PAN@UiO‐66‐NH_2_ sensor. b) Photographic images showcasing the flexibility of the PAN@UiO‐66‐NH_2_ sensor under bending conditions. c) Sensor response to SO_2_ under both flat and bent configurations, demonstrating stability. Reproduced with permission.^[^ ^208^ ^]^ Copyright 2022, Elsevier. d) Schematic of the in‐situ synthesis of large‐area Cu–BHT films using a spin‐coating interfacial self‐assembly technique. e) NH_3_ sensing response of the Cu–BHT‐based sensor under varying bending radii, showcasing its flexibility. Reproduced with permission.^[^ ^209^ ^]^ Copyright 2020, American Chemical Society.

2D MOFs are synthesized via coordination bonds between metal nodes and organic ligands.^[^ ^107^ ^]^ The 2D MOFs nanosheets exhibit ultrahigh surface areas (often 1000–5000 m^2^ g^−1^), diverse pore sizes, high thermal and mechanical stabilities, and versatile structures,^[^ ^206^ ^]^ which make them promising candidates for gas sensing applications.^[^ ^207^ ^]^ For instance, a PANI‐based composite with the MOF UiO‐66‐NH_2_ was synthesized using in situ growth and utilized as the dielectric layer of a capacitive SO_2_ gas sensor at RT (**Figure** **11**a).^[^ ^208^ ^]^ The in situ growth method ensured uniform deposition of UiO‐66‐NH_2_ NPs on the PANI surface while maintaining its porous structure, allowing efficient SO_2_ gas diffusion through the sensor layer. Figure 11b,c demonstrates the fabricated gas sensor with this material with robust bending condition. UiO‐66‐NH_2_, known for its excellent stability, enhanced the sensor's robustness, including washing and bending stability. Although the sensor's response slightly decreased after prolonged washing (24 h), it retained good sensitivity to SO_2_ gas.^[^ ^208^ ^]^

To enhance gas sensing sensitivity, Spieser et al. developed an NH_3_ sensor using a CuBTC/carbon‐graphene composite, which showed a sensitivity 36 times higher than that of a single carbon‐graphene sensor.^[^ ^143^ ^]^ Similarly, He et al.^[^ ^144^ ^]^ developed a flexible and wearable NO gas sensor by integrating Cu(II)‐based MOF Cu–TCA into freestanding TiO_2_ nanochannels. This CuTCA/TiO_2_ p–n heterojunction sensor demonstrated an enhanced gas sensing response, achieving 124% sensitivity to 50 ppm NO. The response and recovery times improved by 25.6% under UV illumination compared to sensing in the absence of light. The improved performance was attributed to the generation and separation of photo‐induced carriers at the Cu–TCA/TiO_2_ heterojunction, which accelerated the sensing kinetics and enhanced the response. Additionally, the selectivity toward NO gas was linked to the reduction of Cu(II) centers to Cu(I) upon electronic interaction between NO molecules and the Cu‐TCA MOF. These findings highlight the potential of CuTCA/TiO_2_‐based sensors for efficient and selective NO detection in wearable applications.

Chen et al.^[^ ^209^ ^]^ reported a facile spin‐coating interfacial self‐assembly technique for the rapid in situ synthesis of copper benzenehexathiol (Cu‐BHT) conductive films, achieving centimeter‐scale fabrication on flexible polyester substrates within just 5 s, as depicted in Figure 11d. The resulting sensor, equipped with a 10 nm‐thick Cu‐BHT film, demonstrated excellent sensitivity to NH_3_ at RT, with a detection limit as low as 0.23 ppm and operating at an ultra‐low voltage of 0.01 V. To evaluate mechanical flexibility, gas sensing responses to 20 ppm NH_3_ were measured under varying bending radii of 10, 7, and 3 mm, as illustrated in Figure 11e. Interestingly, the gas sensing response improved with increased bending, attributed to the formation of microcracks and wrinkles, which provided additional gas adsorption sites. Moreover, the sensor exhibited remarkable mechanical stability, maintaining consistent response and recovery curves even after 1000 bending cycles. This durability highlights the potential of the Cu–BHT sensor for flexible and wearable gas‐sensing applications.

Kun Jo et al.^[^ ^39^ ^]^ proposed a bilayer gas sensor design to improve selectivity, combining MOF NPs with TiO_2_. A mixed matrix membrane (MMM) consisting of zeolitic imidazole framework (ZIF‐7) NPs and polymers was applied as a molecular‐sieving overlayer on a photo‐activated TiO_2_ layer. The ZIF‐7 MMM effectively filtered ethanol and facilitated selective formaldehyde detection through its optimal pore size (≈0.3 nm). This bilayer design demonstrated enhanced selectivity for formaldehyde even in the presence of other gases.^[^ ^39^ ^]^

MOFs are also indirectly used in gas sensor development by deriving sensing materials from MOFs. Wang et al. synthesized CuO from a MOF and fabricated a self‐powered NH_3_ gas sensor using MXene/CuO, powered by a triboelectric nanogenerator.^[^ ^210^ ^]^ This approach exemplifies the potential of MOF‐derived materials in creating advanced flexible and wearable gas sensors.

The advantages are as follows: MOFs offer *unparalleled selectivity* because their pore sizes and chemical functionality can be tailored to target specific gas molecules (sometimes called “molecular sieving” effect). By choosing appropriate metal–ligand combinations, one can design a sensor that is highly sensitive to, say, SO_2_ over CO_2_, as demonstrated by UiO‐66‐NH_2_'s selectivity to SO_2_.^[^ ^3^ ^]^ Flexible MOF sensors benefit from MOFs’ lightweight and the ability to incorporate them into fabrics or thin films (some MOFs can even be grown directly on textiles). Additionally, MOF sensors can often work at RT and show large signal swings because gas adsorption in MOFs significantly changes mass or dielectric properties. The last 5 years have shown that stability issues can be mitigated: Robust MOFs like ZIF‐8 and UiO‐66 have handled humid conditions when embedded in polymers, and some are even washable.^[^ ^3^ ^]^

The limitations are as follows: The *insulating nature* of most MOFs means that pure MOF chemiresistors are rare—one usually needs to add a conductive component (CNTs, graphene, etc.) or use an alternative transduction method (capacitive, quartz crystal microbalance, optical). This can complicate the sensor design. Many MOFs are also sensitive to moisture or heat; water can compete for adsorption sites or even decompose certain MOFs. Thus, for wearable applications, only water‐stable MOFs (or protected composites) are viable. Mechanical brittleness is another issue: MOF crystals are typically micro‐ to nanosized and require a flexible matrix to hold them together in a device—this adds an extra fabrication step. Finally, the long‐term stability under cyclic adsorption/desorption has not been as extensively studied for MOF based gas sensors; some may suffer from gradual pore blocking or framework deformation after repeated exposure to high concentrations of gas.

### 3.7 Hybrid Organic–Inorganic Perovskites Based Flexible Gas Sensor

Hybrid halide perovskites (typically ABX_3_ structures like methylammonium lead iodide, MAPbI_3_, and its variants) are well known in photovoltaics, but they have also shown promise as gas‐sensing materials. These perovskites are semiconductors with ionic charge transport characteristics, and certain gas molecules can reversibly dope or interact with them, leading to sizable electrical changes. Early studies showed that MAPbI_3_ films could detect NH_3_ via a rapid, reversible reaction that changed the film's conductivity and optical absorption.^[^ ^211^, ^212^ ^]^ For flexible sensor development, researchers have exploited the solution‐processability of perovskites to deposit them onto fabrics and papers. *Lead toxicity and stability* are two major concerns for using perovskites in wearable devices, so recent efforts emphasize lead‐free compositions and encapsulation. Maity et al.^[^ ^213^ ^]^ demonstrated a *textile‐based ammonia sensor* using lead‐free methylammonium tin iodide (CH_3_NH_3_SnI_3_) perovskite. They coated this perovskite onto carbon‐fiber cloth and achieved ≈85% resistance change upon exposure to 100 ppm NH_3_. The device operated on only 2 V bias with an output current on the order of nanoamps, indicating ultra‐low power consumption, a key advantage for battery‐powered wearables. While a comparable lead‐based MAPbI_3_ sensor would have higher sensitivity, the CH_3_NH_3_SnI_3_ sensor trades a bit of performance for the benefit of being nontoxic, which the authors note is important for “family life” and IoT applications.^[^ ^214^ ^]^ Importantly, this sensor was part of an e‐textile demonstration, showing that hybrid perovskites can be integrated into clothing for gas detection.

Beyond ammonia, hybrid perovskites have been tested with other gaseslike NO_2_ typically p‐dopes MAPbI_3_ (causing a current increase in a perovskite FET), and some VOCs like dimethylformamide can coordinate with the perovskite and alter its impedance. Additionally in hybrid sensors, chemical redox reactions are commonly employed to convert gas exposure into electrical signals, primarily through resistance changes.^[^ ^215^, ^216^ ^]^

John et al.^[^ ^217^ ^]^ investigated organic–inorganic hybrid perovskite (OIMHP) materials for NO_2_ gas sensing, focusing on improving ambient stability and performance through dimensional engineering. While traditional 3D OIMHP sensors exhibit good room‐temperature sensitivity to NO_2_, their stability is limited. By integrating 2D and 3D structures, the mixed‐dimensional OIMHP sensors achieved significantly enhanced stability, maintaining performance for nearly two months compared to 13 days for the 3D‐only devices. These sensors demonstrated excellent sensitivity (6.3 ± 0.83 times per ppm), rapid response and recovery times (5.7 s and 12.7 s, respectively), and strong selectivity for NO_2_ gas, highlighting their potential for practical, stable, room‐temperature gas sensing applications.

To protect perovskite sensors in wearable scenarios, encapsulation is essential. Encasing the perovskite film with a thin polymer (like parylene or PDMS) can prevent direct contact with moisture while still allowing gas diffusion. Some flexible perovskite sensors have been demonstrated on PI substrates with encapsulation, remaining functional over dozens of bending cycles without degradation in sensing performance.

The advantages are as follows: Hybrid perovskites offer large signal changes (often tens to hundreds of percent) because gas interactions modulate ionic conduction and charge carrier density in the material significantly. They are solution‐deposited at low temperatures, meaning one can coat them onto plastics, paper, or fabrics easily—an inkjet‐printed perovskite sensor array on PI is one envisioned application for e‐nose devices. The response and recovery are fast at RT, and many analytes (NH_3_, NO_2_, amines, and acids) produce distinct responses, which can be leveraged in selective detection (for instance, an array of differently composed perovskites could discriminate gases by their response patterns). Moreover, perovskite sensors can be made low‐power, and some configurations (e.g., photoconductive sensors under light illumination) might even be self‐powered in the future. Mechanically, thin perovskite films (tens of nanometers) are quite flexible and can endure bending when on a compliant substrate, successful tests of perovskite‐on‐paper sensors attest to this.^[^ ^213^ ^]^

The limitations are as follows: *Stability* is the biggest issue—these materials can degrade upon prolonged exposure to air, moisture, or UV light. Without encapsulation, a MAPbI_3_ sensor might lose a significant portion of its sensitivity within days as it converts to PbI_2_. Lead‐based perovskites pose toxicity concerns if they were to leach or deteriorate on a wearable (the amount of lead is small, but regulations may require mitigation). Lead‐free perovskites (e.g., tin‐based) are less toxic but even more chemically unstable (Sn^2^^+^ oxidizes to Sn^4^^+^ easily). Thus, maintaining long‐term performance is challenging. Reproducibility is another factor: Perovskite films can vary in crystallinity and grain boundaries, which affect sensor behavior; careful fabrication control is needed for consistent devices. Finally, perovskites are sensitive to both target gases and interfering gases (and humidity), so ensuring selectivity may require either filtering (which is not trivial in a wearable) or using sensor arrays with machine learning to differentiate responses. Despite these challenges, the ongoing progress in stabilizing perovskites and the demonstration of wearable formats indicate a growing viability of this class of materials in practical gas sensing.

The gas sensing capabilities of all other 2D materials‐based flexible and wearable gas sensors except TMDs are comprehensively summarized in Table 4. These sensors demonstrate versatility in detecting various gases, showcasing their applicability for environmental monitoring and healthcare. Notably, most of these sensors maintain their functionality and sensitivity under dynamic mechanical conditions, such as bending, twisting, and stretching. This resilience highlights the adaptability of 2D material‐based sensors in real‐world wearable applications, reinforcing their potential for integration into flexible electronics and IoT systems. By leveraging their inherent mechanical flexibility and high sensitivity, these sensors are positioned as pivotal technologies in the development of advanced, user‐friendly gas detection systems.

### 3.8 Applications for Wearable Gas Sensors: Linking Target Gases with 2D Materials

Wearable gas sensors based on 2D materials have potential applications across diverse fields. The most prominent areas include environmental air quality monitoring, healthcare monitoring (via exhaled breath analysis or skin‐emitted gas detection), and safety in industrial or domestic settings (hazardous gas alarms). Different applications prioritize different target gases and performance requirements, and particular 2D material systems can be identified as especially suitable for each use case.

#### 3.8.1 Environmental Monitoring

For environmental air‐quality monitoring, target gases include urban pollutants (e.g., NO_2_, O_3_, and SO_2_), greenhouse or fuel gases (CH_4_ and CO), and harmful VOCs like formaldehyde or benzene.^[^ ^218^ ^]^ These applications demand ultrahigh sensitivity (often ppb‐level) and stability under ambient conditions. 2D material hybrids that maximize surface area and catalytic adsorption are therefore favored. For example, metal oxide‐functionalized 2D materials and MOF‐on‐2D hybrids have achieved exceptional low detection limits. A recent study demonstrated a LIG platform coated with a 2D copper MOF for NO_2_, achieving one of the fastest responses (≈16 s) and a sub‐ppb LOD (≈0.17 ppb) at RT.^[^ ^219^ ^]^ Such a graphene/MOF hybrid exploits the MOF's porous adsorption and the conductive 2D network to detect trace pollutants. Similarly, decorating graphene or MXene sheets with metal oxides (e.g., SnO_2_ and ZnO) provides abundant reactive sites for gas adsorption, enabling ppb‐level sensing of oxidizing gases.^[^ ^218^ ^]^ These composites leverage the oxide's catalytic affinity for gases like NO_2_ or H_2_S and the 2D material's high charge mobility to transduce low‐concentration adsorption events into electrical signals. Overall, 2D hybrids for environmental monitoring prioritize maximum surface interaction, selectivity to target pollutants, and operation at low power (RT) for long‐term outdoor use.^[^ ^219^, ^220^ ^]^

#### 3.8.2 Healthcare and Wearables

Wearable gas sensors for healthcare focus on biocompatible, skin‐, or breath‐contact devices that can detect biomarkers and environmental exposures relevant to personal health. Key targets include exhaled breath biomarkers such as acetone (diabetes monitoring), ammonia (indicator of liver/kidney function), nitric oxide (inflammation/asthma), ethanol (metabolic byproduct), hydrogen sulfide (halitosis), and other VOCs linked to disease.^[^ ^221^, ^222^ ^]^ In this arena, 2D material systems must be flexible, nontoxic, and highly selective, since human breath or skin emissions are complex mixtures. Graphene‐based composites are prominent due to graphene's excellent flexibility, conductivity, and inherent biocompatibility.^[^ ^3^ ^]^ Selectivity is enhanced by functionalizing 2D materials with polymers or biomolecular receptors. For instance, a polypyrrole‐sulfonated graphene composite was recently reported as an ultrasensitive, wearable ammonia sensor for noninvasive health monitoring. This flexible nanocomposite sensor could detect breath ammonia from ≈0.5 ppb to tens of ppm, with fast response and high specificity against common breath interferents.^[^ ^221^ ^]^ The sulfonated graphene provides a high‐surface‐area conductive scaffold, while the polypyrrole contributes selective affinity to NH_3_, together enabling ppb‐level sensing at near‐body temperature. Similar graphene/polymer or graphene/MOF hybrids have been explored for acetone and NO detection in breath, offering real‐time monitoring capabilities in wearable formats.^[^ ^220^ ^]^ In all cases, the materials are chosen to be lightweight and skin‐friendly (e.g., printable graphene inks, MXene films) and to maintain stability in humid, warm conditions. High selectivity (to differentiate specific biomarkers), rapid recovery, and integration with wearable substrates (textiles, bandages, and masks) are key requirements for 2D‐material‐based healthcare sensors.^[^ ^3^, ^221^ ^]^

#### 3.8.3 Industrial Safety

Industrial safety applications require robust wearable sensors for the early detection of toxic or explosive gases in workplaces. Target gases include CO and H_2_S (toxic gases in confined spaces and oil/gas industry), ammonia or chlorine (chemical leaks), and flammable gases like H_2_ and CH_4_.^[^ ^218^, ^220^ ^]^ These scenarios prioritize fast response/recovery times and reliability over a wide concentration range, as sensors often serve as alarm systems for worker protection. 2D materials decorated with catalytically active noble metal NPs have emerged as ideal sensing systems for this purpose. Noble metals (e.g., Pt, Pd, and Au) on 2D semiconductors (such as MoS_2_, SnS_2_, or graphene) create active sites that facilitate gas reactions at RT, dramatically boosting response speed and sensitivity.^[^ ^223^ ^]^ For example, Pd‐decorated MoS_2_ sensors show significantly enhanced H_2_ response versus pristine MoS_2_, attributed to Pd's catalytic dissociation of H_2_ which induces greater charge transfer in the 2D layer. Likewise, Au or Ag NPs on layered materials can chemisorb sulfur‐containing gases (like H_2_S), accelerating reaction kinetics and improving selectivity to those targets.^[^ ^223^ ^]^ Metal‐decorated 2D heterostructures thus achieve the rapid, ppm‐level detection needed for leak alarms (often reacting in seconds). In addition, hybrid 2D/metal‐oxide systems are used in industrial sensors to combine the 2D material's flexibility with the metal oxide's robustness. A composite of rGO with SnO_2_, for instance, exhibited higher NO_2_ response and faster recovery than graphene alone, due to SnO_2_ providing extra active sites for gas adsorption.^[^ ^218^ ^]^ For wearable safety devices, the chosen 2D material systems must also endure harsh conditions (temperature fluctuations, humidity, and continuous operation) while minimizing power consumption. By leveraging catalytic 2D hybrids, modern wearable sensors can meet industrial requirements for immediacy, sensitivity, and stability in detecting life‐threatening gas hazards.^[^ ^223^ ^]^

**Table** **5** summarizes key application areas, representative target gases, recommended 2D material‐based sensing systems (with rationale), and key performance requirements. Each application area thus benefits from a tailored 2D material approach. Environmental monitors leverage high‐surface‐area 2D hybrids for ppb‐level sensitivity to pollutants, wearable health sensors employ graphene and similar flexible composites to achieve biocompatible and selective detection of biomarkers, and industrial safety devices incorporate catalytically enhanced 2D systems (often with noble metals) for fast, reliable detection of dangerous gases. This targeted pairing of gas analytes with optimized 2D material systems provides a framework for designing next‐generation wearable gas sensors that meet the stringent requirements of real‐world applications. The strategic selection of 2D materials and composites based on the intended environment and gas target is a novel paradigm that not only addresses current performance gaps but also offers a roadmap for future innovations in wearable sensing technology.

**Table 5:** Application‐specific target gases and optimal 2D material systems for wearable gas sensors, with rationale, key requirements, and supporting references.

Application area	Target gases (examples)	Recommended 2D materials system and rationale	Key requirements
Environmental monitoring	NO_2_, O_3_, SO_2_ (urban air pollutants); CO; CH_4_; H_2_S; VOCs (e.g., formaldehyde, benzene) at ppb–ppm levels.	*Metal oxide‐functionalized 2D materials* (e.g., SnO_2_‐graphene, ZnO–MoS_2_): metal oxides provide catalytic adsorption sites and amplify gas‐induced charge transfer, while the 2D matrix offers high conductivity and large surface area. *MOF* *‐on‐2D hybrids*(e.g., 2D MOF on graphene or MXene): MOFs’ nanoporous networks trap trace gases, and underlying 2D conductors transduce these interactions, enabling detection of sub‐ppm pollutants. These hybrids achieve ultralow LODs (often <1 ppb) and enhanced selectivity by tailoring pore chemistry to specific gases	– Ultrahigh sensitivity (ppb‐level detection) at ambient temperature – Selectivity to target gas over background (e.g., distinguish NO_2_ among VOCs) – Stability under varying humidity and temperature (outdoor conditions) – Low power consumption for continuous air monitoring
Healthcare and wearables	Breath biomarkers and personal exposure gases: acetone (diabetes), NH_3_ (kidney/liver health), NO (airway inflammation), H_2_S (halitosis), ethanol and isoprene (metabolic markers), etc., typically in ppb–ppm range	*Graphene‐based composites* (e.g., graphene with conducting polymer or biomolecule coatings): graphene provides a flexible, biocompatible sensor platform, while functionalization (polymers, enzymes, or MOFs) imparts selectivity to specific biomarker gases *Example:* A polypyrrole‐sulfonated graphene sensor achieves selective NH_3_ detection in breath (down to sub‐ppb) for noninvasive health monitoring. *2D MXene or* *TMD* *hybrids* (e.g., MXene/metal oxide nanocomposites): combine high surface area and hydrophilicity (for breath humidity tolerance) with tunable surface chemistry for selective acetone or NO sensing. These systems can be integrated into wearable substrates (e.g., facemasks, skin patches) to continuously track health‐indicative gases	– Biocompatibility and skin/breath safety (nontoxic, breathable sensor layers) – Flexibility and stretchability for on‐body or in‐clothing devices – High selectivity to target biomarkers in complex mixtures (resist interference from humidity, VOCs) – Low detection limit at or below biomarker threshold levels (ppb–low ppm) – Low‐power operation at near body temperature
Industrial safety	Toxic gases (CO, H_2_S, NH_3_, Cl_2_, and NO_2_) and flammable gases (H_2_, CH_4_, and volatile organics) in workplaces; concentrations from ppm leaks to high levels in accidents	*Noble metal‐decorated 2D materials* (e.g., Pd or Pt NP‐decorated MoS_2_, graphene, or MXene): noble metals catalyze the reaction or dissociation of target gases (H_2_, CO, etc.) on the sensor surface, yielding a rapid and large resistance change. This yields ultrafast responses and lower operating temperatures compared to undoped sensors *Example:* Pd–MoS_2_ hybrids show room‐temperature H_2_ sensing with greatly enhanced response speed and sensitivity, as Pd captures and splits H_2_ molecules. *2D material/metal oxide core–shells* (e.g., rGO coated with SnO_2_ or CuO): marry the flexibility of 2D films with the robustness and high signal of metal oxides, useful for detecting acids or sulfides in harsh industrial environments. These hybrid systems improve reliability and recovery in real‐world conditions	– Fast response and recovery times (seconds) for early leak detection alarms – Wide dynamic range (from sub‐ppm to high ppm or % levels) to handle both trace leaks and concentrated releases – Stability and repeatability under extreme conditions (temperature swings, high humidity, presence of multiple gases) – Portability and wireless integration (for personal safety gear) – Intrinsically safe operation (RT sensing to avoid ignition sources)

## 4 Current Limitations, and Challenges of 2D Materials‐Based Flexible and Wearable Gas Sensors

Real‐world deployment of flexible and wearable 2D material gas sensors for ambient air monitoring is impeded by several practical challenges. Key issues that must be addressed include the following.

### 4.1 Long‐Term Stability

Prolonged exposure to ambient conditions can deteriorate 2D materials, leading to loss of sensor performance over time. Even chemically robust 2D structures are prone to gradual oxidation, adsorption of airborne contaminants, or structural changes. For instance, unprotected graphene and MoS_2_ films show notable degradation when stored in air, with oxidation and moisture uptake altering their electrical properties and baseline signals.^[^ ^224^ ^]^ Emerging 2D materials like MXenes (e.g., Ti_3_C_2_T_x_) can also oxidize within a few weeks of exposure, dramatically reducing their conductivity and gas response.^[^ ^189^ ^]^ To combat such aging, encapsulation strategies have been explored—depositing a thin Al_2_O_3_ passivation layer over the 2D sensing film can significantly slow down environmental degradation by blocking oxygen and water ingress.^[^ ^225^ ^]^ However, while encapsulation preserves the material, it may also impede gas diffusion, highlighting the need for protective coatings that balance stability and sensor sensitivity.

### 4.2 Signal Degradation (Drift)

2D material sensors often exhibit signal drift—a gradual change in baseline resistance or sensitivity during continuous operation or over repeated sensing cycles. This drift arises from slow chemisorption of gas molecules, residual charges in the material, or microstructural changes in the sensing layer. In an outdoor application, such baseline wander can lead to false readings or require frequent recalibration to maintain accuracy. Mitigating drift is challenging; nonetheless, researchers are developing quantitative predictive frameworks to correct sensor drift over time, using models or machine learning to forecast and compensate for baseline shifts.^[^ ^132^ ^]^ These approaches allow the sensor's output to be adjusted in software, extending its usable lifetime without physical intervention. Combining material improvements (to reduce inherent drift) with algorithmic drift compensation is an important strategy for reliable long‐term monitoring.

### 4.3 Ambient Interference (Humidity and Temperature)

Flexible 2D gas sensors operating in ambient air must contend with fluctuating humidity and temperature, which can significantly interfere with sensor signals. Many 2D materials are inherently sensitive to water vapor—for example, graphene and its derivatives readily adsorb moisture, leading to changes in conductance that can mask the response to target gases. Similarly, temperature variations alter charge carrier mobility and adsorption kinetics on 2D surfaces, causing output fluctuations unrelated to gas concentration. In real‐world conditions, this means a sensor's readings might drift with the weather or the wearer's perspiration and body heat. To address humidity interference, surface functionalization and material modifications have been employed. A notable example is BP, a 2D material that normally degrades rapidly in humid air; researchers demonstrated that fluorinating or coating BP can render it amphiphobic, enabling stable sensor operation even at ≈95% relative humidity for months.^[^ ^226^ ^]^ Temperature compensation circuits and reference sensors are also integrated in some designs to normalize the gas readings. Nonetheless, achieving selectivity against ambient factors remains a critical challenge for accurate gas detection in uncontrolled environments.

### 4.4 Device‐to‐Device Reproducibility

Achieving consistent performance across multiple 2D material sensors is difficult, especially for wearable and printed devices produced in large batches. Minor variations in 2D material synthesis (flake size, defect density, and surface chemistry) or in fabrication processes (printing uniformity, layer thickness, and electrode contacts) can lead each nominally identical sensor to exhibit different baseline resistances or sensitivities. In practical deployments—for example, distributed wearable air‐quality badges—such device‐to‐device variability complicates calibration and reliability. Large‐scale fabrication methods like R2R printing and screen printing have been applied to fabricate graphene‐based gas sensor arrays on flexible substrates, but ensuring uniform gas response and low parameter spread between devices remains challenging.^[^ ^148^ ^]^ For instance, printed graphene sensors can show variance in response due to inhomogeneous ink deposition or drying patterns, requiring individual calibration.^[^ ^3^ ^]^ Improving reproducibility may involve better control of material quality (e.g., using highly consistent graphene inks or well‐defined 2D material formulations) and process refinements such as printing at optimized settings or post‐print annealing. Ultimately, standardizing fabrication and incorporating on‐chip calibration elements will be necessary to attain reliable, reproducible performance at scale.

### 4.5 Performance under Continuous Operation

Continuous, long‐duration operation of gas sensors is expected in real‐world monitoring (e.g., an air quality sensor that runs 24/7), yet many 2D material sensors experience performance degradation when operated nonstop. Extended exposure to target gases or continuous electrical bias can lead to sensor “fatigue”—active sites on the 2D material become saturated or poisoned by irreversibly bound molecules, and the sensor's response gradually declines. Additionally, constant operation can accelerate processes like thermal stress or slow oxidation, further diminishing sensitivity. As a result, a sensor might initially detect a gas well but fail to maintain the same response after hours or days of continuous use. One emerging solution is the development of self‐healing sensor systems that can rejuvenate their sensing surface. For example, researchers have created self‐healing conductive composites for gas sensors that can restore their electrical and sensing properties after being damaged or fouled.^[^ ^152^ ^]^ These materials use dynamic bonds or reversible interactions, allowing the sensor film to “heal” microcracks or desorb accumulated species when given a mild stimulus (such as heat or rest), thereby recovering performance. Periodic regeneration protocols (e.g., UV or thermal cleaning cycles) are also employed in some 2D sensor designs to refresh the surface. While these approaches improve durability, designing sensors that inherently resist degradation under continuous operation—or can autonomously recover—is a key goal for future wearable gas monitoring devices.

## 5 Conclusions and Future Prospects

The rapid evolution of 2D‐material‐based flexible and wearable gas sensors underscores their growing relevance in fields such as environmental monitoring, healthcare diagnostics, and industrial safety. This review has critically analyzed diverse material classes, sensor architectures, and fabrication methods, emphasizing how application‐specific requirements shape material choices. While impressive strides have been made, key barriers remain before these technologies can be fully realized in practical, real‐world deployments.

Future progress will hinge on solving interrelated challenges of material degradation, signal drift, environmental interference, and production scalability. One promising direction is the rational design of multifunctional hybrid materials, where synergistic interactions such as combining catalytic metal NPs with conductive 2D sheets can enhance both sensitivity and selectivity without compromising flexibility. Similarly, the use of bio‐inspired and adaptive sensing interfaces, capable of dynamically responding to environmental fluctuations, holds potential for improving robustness in wearable formats.

From a system‐level perspective, next‐generation sensors must incorporate on‐board intelligence to process and interpret signals reliably. Integration of lightweight electronics with machine learning‐based algorithms can enable real‐time classification of complex gas mixtures and automated drift correction, especially in wearable health applications. Emerging technologies such as neuromorphic sensors, where sensing and computation co‐exist on a single flexible platform, offer intriguing possibilities for low‐power, real‐time analysis.

Moreover, attention must shift toward scalable and reproducible manufacturing. While methods like inkjet printing, screen printing, and R2R deposition have demonstrated promise, further work is needed to ensure high‐yield fabrication of uniform, defect‐free films across large areas. Development of sensor arrays or multiplexed platforms, rather than standalone devices, will also enable multi‐analyte detection and improve diagnostic accuracy in applications such as breath analysis or air quality monitoring.

Materials that exhibit self‐repairing capabilities or stimuli‐responsive properties such as self‐healing polymers or thermally adaptive substrates could improve long‐term performance and device lifetime. Meanwhile, green synthesis approaches and biodegradable substrates are expected to become increasingly important in the context of sustainability and disposable medical diagnostics.

In conclusion, while 2D materials have already demonstrated remarkable promise for flexible and wearable gas sensors, the path forward demands a multidisciplinary effort that blends materials innovation, smart integration, and system‐level design. Strategic research into heterostructure engineering, sensor fusion, and real‐world validation will be crucial to elevate these sensors from lab prototypes to impactful, commercial‐ready technologies. With continued innovation, such sensors are poised to play a transformative role in enabling decentralized diagnostics, personal environmental exposure tracking, and responsive safety systems in the era of the IoT.

## Conflict of Interest

The authors declare no conflict of interest.
